# Cdc42 Deficiency Leads To Epidermal Barrier Dysfunction by Regulating Intercellular Junctions and Keratinization of Epidermal Cells during Mouse Skin Development

**DOI:** 10.7150/thno.34014

**Published:** 2019-07-09

**Authors:** Min Zhang, Xueer Wang, Fukun Guo, Qin Jia, Nuyun Liu, Yinghua Chen, Yuan Yan, Mianbo Huang, Huiyi Tang, Ying Deng, Simin Huang, Zhitao Zhou, Lu Zhang, Lin Zhang

**Affiliations:** 1 Department of Histology and Embryology, Guangdong Provincial Key Laboratory of Tissue Construction and Detection, School of Basic Medical Sciences, Southern Medical University, Guangzhou, People's Republic of China; 2 Guangdong Provincial Key Laboratory of Proteomics, Key Laboratory of Mental Health of the Ministry of Education, School of Basic Medical Sciences, Southern Medical University, Guangzhou, People's Republic of China; 3 Division of Experimental Hematology and Cancer Biology, Children's Hospital Research Foundation, Cincinnati, Ohio, USA; 4 Institute of Comparative Medicine & Laboratory Animal Center, Southern Medical University, Guangzhou, 510515, China.

**Keywords:** Cdc42, SPRR, epidermal barrier, epidermal development

## Abstract

**Rationale**: Cdc42 is a Rho GTPase that regulates diverse cellular functions. Here, we used genetic techniques to investigate the role of Cdc42 in epidermal development and epidermal barrier formation.

**Methods**: Keratinocyte-restricted Cdc42 knockout mice were generated with the Cre-LoxP system under the keratin 14 (K14) promoter. The skin and other tissues were collected from mutant and wild-type mice, and their cellular, molecular, morphological, and physiological features were analyzed.

**Results**: Loss of Cdc42 in the epidermis *in vivo* resulted in neonatal lethality and impairment of epidermal barrier formation. Cdc42 deficiency led to the loss of epidermal stem cells. The absence of Cdc42 led to increased thickening of the epidermis, which was associated with increased proliferation and reduced apoptosis of keratinocytes. In addition, Cdc42 deficiency damaged tight junctions, adherens junctions and desmosomes. RNA sequencing results showed that the most significantly altered genes were enriched by the terms of “keratinization” and “cornified envelope” (CE). Among the differentially expressed genes in the CE term, several members of the small proline-rich protein (SPRR) family were upregulated. Further study revealed that there may be a Cdc42-SPRR pathway, which may correlate with epidermal barrier function.

**Conclusions**: Our study indicates that Cdc42 is essential for epidermal development and epidermal barrier formation. Defects in Cdc42-SPRR signaling may be associated with skin barrier dysfunction and a variety of skin diseases.

## Introduction

The epidermis, as the outer layer of the skin, serves as the primary barrier protecting the organism against major environmental stresses 1. There are two central aspects of epidermal barrier function: the outside-in barrier, which hinders the infiltration of external materials into the epidermis, and the inside-out barrier, which prevents the leakage of water and electrolytes from the epidermis 2, 3. Defects in epidermal barrier formation during embryonic development present challenges to the neonate and even result in neonatal lethality 3, 4.

The epidermis originates from a single layer of multipotent embryonic progenitor keratinocytes. During embryonic development, epidermal stem cells undergo a particular differentiation program and generate a stratified epidermis 5. The epidermal stem cells in the basal layer maintain a balance between quiescence and proliferation 6. Keratin 5 (K5) and keratin 14 (K14) are the main structural proteins within the basal keratinocytes 7. After commitment to differentiation, epidermal stem cells migrate from the stratum basale (SB) into the stratum spinosum (SS), and the spinous cells progressively lose their proliferative potential 8. As cells progress to the stratum granulosum (SG), cornified envelope proteins, such as involucrin, loricrin and small proline-rich proteins (SPRRs), are synthesized 9. Cornified envelopes are produced in the final step of terminal differentiation; these structures are composed of highly cross-linked lipids and proteins that seal the epidermis to create the outside-in barrier in the stratum corneum (SC) 10, 11. The importance of intercellular junction proteins in inside-out barrier formation has been highlighted by genetic studies of junction proteins 12. However, the mechanisms controlling skin morphogenesis and epidermal barrier formation are still poorly understood.

Cdc42 is a member of the Rho GTPase family of intracellular switches that regulate multiple signaling pathways involved in actomyosin organization and cell polarity 13, 14. Cdc42 knockout (KO) is embryonic lethal in mice, with the affected animals dying before embryonic day 7.5; thus, tissue-specific Cdc42 KO models have been developed to study Cdc42 function beyond early embryogenesis 15, 16. To analyze the function of Cdc42 in skin, Wu et al. generated mice with a keratinocyte-restricted deletion of the *Cdc42* gene under the K5 promoter (Cdc42fl/fl K5) 17. The mutant offspring were born without obvious defects. However, within four weeks, the mutant mice lost all their hair and did not regrow it. The investigators demonstrated that a reduction in cytoplasmic β-catenin leads to rapid loss of nuclear β-catenin in keratinocyte stem cells and impaired hair follicle differentiation 17. However, the role of Cdc42 in epidermal development and barrier formation was not elucidated.

In this study, we independently generated keratinocyte-restricted Cdc42 knockout mice with the Cre-LoxP system under the K14 promoter to investigate the role of Cdc42 in mouse epidermal development. Unexpectedly, the Cdc42 conditional KO mice died of epidermal barrier impairment within 1 day after birth. We found that Cdc42 deletion dampens both outside-in and inside-out barrier function during epidermal development. In addition, we demonstrated that Cdc42 plays crucial roles in regulating the balance between keratinocyte proliferation and differentiation and in the integrity of cell-cell junctions in epidermal development.

## Materials & Methods

### Primary antibodies

Primary antibodies against the following antigens were used: K1, K14, K15, K19, E-cadherin, Cdc42, AP-1, p-PAK1 (Abcam, UK), K6, loricrin, involucrin (Covance, USA), β-catenin, bromodeoxyuridine (BrdU; Santa Cruz, USA), α6-integrin (BD Biotech, USA), F4/80 (eBioscience, USA), SPRR1A (Biorbyt, UK), SPRR1B (Aviva Systems Biology, USA), desmoplakin, ZO-1, SPRR2D, PAK1, cyclin D1, caspase 3, occludin and SPRR2G (Proteintech, China), Bim, p-Bim, Bcl-2, p-Bcl-2, JNK, p-JNK, p38, p-p38, ERK, and p-ERK (Cell Signaling, USA).

### Animals

Conditional targeted Cdc42^flox/flox^ mice were generated as described by Melendez et al.^18^. Cdc42^flox/flox^-K14-Cre^+^ mice were generated as follows. In order to produce Cdc42 conditional KO mice, the guanine nucleotide binding sequence encoding exon 2 of the Cdc42 gene was flanked by a pair of loxP sequences. In order to delete Cdc42 *in vivo* in the epidermis, the Cdc42^flox/flox^ mice were mated with mice expressing Cre recombinase under the control of the K14 proximal promoter (The Jackson Laboratory, USA). The primer sequences are listed in the Supplementary Materials and Methods.

Animals were housed under specific pathogen- free conditions in the animal facility at Southern Medical University Laboratory Animal Center Research Foundation. All animal studies were approved by the Bioethics Committee of Southern Medical University.

### Functional analyses of the epidermal barrier and skin permeability of mouse embryos

In order to determine the rate of fluid loss, newborn animals were separated from their mothers and placed on a heating pad set at 37 °C. Body weight was monitored every 60 min until the homozygous mutant mice died. Transepidermal water loss (TEWL) was measured using an Aquaflux AF-200 Tewameter (Courage and Khazaka, UK). Dye exclusion assays were performed as described previously 19. Unfixed embryos were immersed in a low-pH X-gal substrate solution at 30-37 °C for several hours to overnight until the color developed. The tails were removed with scissors to serve as a positive control for staining.

### Toluidine blue penetration assay and tight junction (TJ) permeability assay

An *in situ* skin permeability assay using toluidine blue was performed as reported by Hardman et al. 19. The TJ permeability assay was performed as reported by Furuse et al. 20. Fifty microliters of 10 mg/ml EZ-Link Sulfo-NHS-LC-Biotin (Thermo Fisher Scientific, USA) in phosphate-buffered saline (PBS) containing 1 mM CaCl_2_ was injected into the dermis on the backs of the Cdc42 KO and wild-type (WT) newborns. After a 30-min incubation period, the skin was removed and frozen. Approximately 5 μm-thick frozen sections were fixed in 95% ethanol at 4 °C for 30 min and then in 100% acetone at room temperature for 1 min. The sections were soaked in blocking solution for 15 min, incubated with anti-occludin for 30 min, washed three times with blocking solution, and then incubated with a mixture of DyLight 48 anti-rabbit IgG pAb (Thermo Fisher Scientific, USA) and streptavidin-conjugated Alexa Fluor™ 594 (Invitrogen, USA) for 30 min.

### Embryos and histological examination

For timed pregnancy, Cdc42flox/flox-K14-Cre- female mice were mated with Cdc42flox/wt-K14-Cre+ male mice from 4:00 to 8:00 p.m. The next day, the males and females were separated. At 8:00 a.m., if a vaginal plug was detected, the gestational age of the offspring was considered embryonic day (E) 0.5 post coitum (E0.5). E14.5-E18.5 fetal mice and P1 animals were killed by terminal inhalation anesthesia. The tail tips were removed for genotyping. The mice were fixed in 4% paraformaldehyde, dehydrated with a graded ethanol series, cleared in dimethylbenzene, and embedded in paraffin. The tissue was sectioned into 5-μm sections, which were deparaffinized by immersion in dimethylbenzene and then rehydrated. Hematoxylin and eosin (H&E) staining was performed according to standard procedures.

### Electron microscopy analysis

To prepare samples for transmission electron microscopy (TEM), the full-thickness dorsal skin (1× 1 × 1 mm) of E17.5 and E19.5 fetal mice was harvested on ice, fixed with 2.5% glutaraldehyde and then postfixed with osmic acid. Sections were stained, and images were obtained under an H-7500 transmission electron microscope (Hitachi Science Systems, Ltd., Japan). In order to prepare samples for scanning electron microscopy (SEM), the full-thickness dorsal skin of E17.5 and postnatal day (P) 1 fetal mice was harvested on ice and then fixed with 2.5% glutaraldehyde. Spray-dried samples were analyzed by SEM (S-3000-N, Hitachi Science Systems, Ltd., Japan) 21.

### Immunohistochemistry and immunofluorescence

Briefly, tissue sections were heated in citrate buffer. After being blocked, the sections were immunostained with primary antibodies. Then, the sections were incubated with biotinylated secondary antibodies followed by avidin-biotin-peroxidase complex and 3,3'-diaminobenzidine (DAB) reagent (Santa Cruz, USA), or they were incubated with secondary antibodies labeled with Alexa Fluor 546 (Invitrogen, USA) and were also stained with Hoechst 33342 (Sigma, USA). Subsequently, all sections were visualized under a microscope (DM40008, Leica, Germany). Images were captured using Leica Application Suite 3.7.

### *In vivo* cell proliferation and apoptosis assays

Fetal mice were injected with BrdU solution (Sigma, USA, 20 mg/ml, dissolved in 0.1 M PBS) intraperitoneally (200 mg/kg body mass). Two hours later, the E15.5 to E18.5 fetal mice were removed. Cell proliferation was determined by counting the number of BrdU-positive cells per 100 μm in the epidermis after BrdU immunohistochemistry. Cell apoptosis was detected by terminal deoxynucleotidyl transferase dUTP nick end labeling (TUNEL) using an apoptosis cell detection kit (Trevigen, USA). The number of TUNEL-positive cells was recorded using the above approach. Detailed methods for these assays have been described previously 22.

### Primary keratinocyte culture, proliferation and apoptosis assays

Mouse primary keratinocytes were isolated from newborn fetuses, as described previously 23. The cells were then incubated with primary K15 or K19 antibody. Next, the cells were incubated with secondary antibodies labeled with Alexa Fluor 546 and Hoechst 33342, as described previously 24.

Mouse primary keratinocytes were isolated from Cdc42^loxp/loxp-Cre-^ neonates. The cells were then infected with viruses (vector, plenti-CMV-NLS-Cre (plenti-Cre), 1 × 10^9^ infectious units/ml, purchased from OBiO Technology (Shanghai) Corp., Ltd.); after 3 days, the expression of Cdc42 was detected by Western blotting (WB). Then, the primary keratinocytes were cultured in high-calcium medium (Ca^2+^ concentration increased from 0.09 to 2 mM) for cell experiments (cell-cell junctions and SPRR family members) as described previously 25.

The proliferation of primary keratinocytes was detected with a 5-ethyl-2′-deoxyuridine (EdU) assay (Cell-Light EdU Apollo 567 In Vitro Kit, Ribobio, China). Apoptotic primary keratinocytes were detected using a TUNEL BrightRed Apoptosis Detection Kit (Vazyme Biotech Co., Ltd., China).

### RNA sequencing, analysis of differentially expressed genes, and Gene Ontology (GO) and pathway analysis

Sequencing was performed using an Illumina Genome Analyzer (HiSeq 2000). Procedures are described in the Supplementary Materials and Methods.

### Cdc42N17 lentivirus, SPRR1B lentivirus, infection and pull-down assay

The lentiviral overexpression vector pLV[Exp-EGFP/Neo-CMV>mSprr1b[ORF045957] and interference vector pLV[shRNA]-EGFP/Neo-U6>mSprr1b [shRNA] were purchased from Cyagen Biotechnology Co., Ltd., renamed mSprr1b[ORF045957] and mSprr1b[shRNA], respectively. The lentivirus-bearing empty vector and dominant negative mutant of Cdc42 (Cdc42N17) were purchased from Obio Technology (Shanghai) Corp, Ltd. All viral preparations were titrated to contain 1 × 10^8^ infectious units/ml. C57BL/6 mice were anesthetized with an intraperitoneal injection of 1% pentobarbital sodium (Sigma; w/v; 0.01 ml/g body mass). The hair was shorn from the dorsal skin of 7-week-old mice using curved scissors (2.5 × 2.5 cm area), after which a honey-and-wax mixture (Mayllice, Italian CP-WAX Corporation) was applied to the area. All of the dorsal skin hair follicles were in the telogen stage, as evidenced by the homogeneous pink skin color. After removal of the honey-and-wax mixture, 0.02 ml of viral vector at a titer of 1 × 10^8^ infectious units/ml was applied to the surface of the skin (0.5 × 0.5 cm area). Another 0.02 ml of the viral vector was injected subcutaneously. The infection efficiency was determined by examining green fluorescence in tissue sections with a fluorescence microscope three days after lentiviral infection. Cdc42 activity (Cdc42-GTP) was assessed with a GTPase pull-down assay three days after lentiviral infection. The GTPase pull-down assay was performed as previously described 26. After the keratinocytes were grown to 50% confluency in medium, 0.002 ml of viral vector per well was applied to the keratinocytes in a 24-well plate. Once again, the infection efficiency was determined by examining green fluorescence.

### Quantitative real-time polymerase chain reaction (qRT-PCR) validation

qRT-PCR was performed as previously described 26. Each biological replicate (E15.5 WT [n = 8], E15.5 KO [n = 8], E17.5 WT [n = 8], E17.5 KO [n = 8], P1 WT [n = 8] and P1 KO [n = 8]) was run in triplicate to monitor technical variability and provide quality control. For data normalization across samples, the housekeeping gene glyceraldehyde 3-phosphate dehydrogenase (GAPDH) was used as an endogenous control gene. Primers for all the tested differentially expressed genes (DEGs) and GAPDH are listed in Supplementary Table 1. Normalization of threshold cycle (Ct) values for each gene and determination of fold differences in gene expression (normalized to WT mice) were conducted by the 2^-ΔΔCt^ method. Statistical analyses were performed using the two-sample t-test and one-way analysis of variance (ANOVA) followed by the Newman-Keuls multiple comparison test using GraphPad Prism 5.0 (GraphPad Software, USA).

### Synthesis and intradermal injection of siRNA against SPRR2D

The mouse SPRR2D subunit sequence was obtained from GenBank (Gene ID: 20758). Sequence- specific siRNAs targeting mouse SPRR2D were synthesized by RiboBio Co., Ltd. Meanwhile, negative control siRNA (NC) was designed as a negative control. The siRNA sequence was as follows: GAACTATAGCTGCTATCTA. A volume of 50 ml PBS containing 20 mg siRNA and 2 mg of pL2G plasmid was injected into the skin of the mice once a day for 3 days. The same amount of NC siRNA was applied as a negative control. Then, SPRR2D expression was detected by WB** (Figure S3I).** The injected area was sampled for histology and RT-PCR analysis.

### Statistical analysis

Data were analyzed using IBM SPSS Statistics 20 software and plotted as the mean ± standard deviation. The two-sample t-test and one-way ANOVA were used to identify statistically significant differences.

## Results

### Generation and gross phenotypes of keratinocyte-restricted Cdc42 knockout mice

We generated Cdc42 conditional KO mice with the Cre-LoxP recombination system under the control of the K14 gene promoter **(Figure S1A)**. In these mice, K14 activates Cre recombinase expression in the epidermis at E14.5 27. PCR was used to confirm that the Cdc42 alleles were knocked out** (Figure S1B)**. From E15.5 to P1, the quantity of Cdc42 protein in the epidermis gradually declined in the KO mice compared to their WT littermate controls **(Figure S1C)**, and Cdc42 protein was entirely absent from the epidermis of P1 KO mice **(Figure S1D)**. Furthermore, the immunohistochemistry, PCR and WB results showed that Cdc42 was not changed in the stomach, intestine, lung, liver or heart of Cdc42 KO mice but was deleted from the esophagus, which contains K14-positive cells **(Figure S1E-G)**.

**Figure 1:**
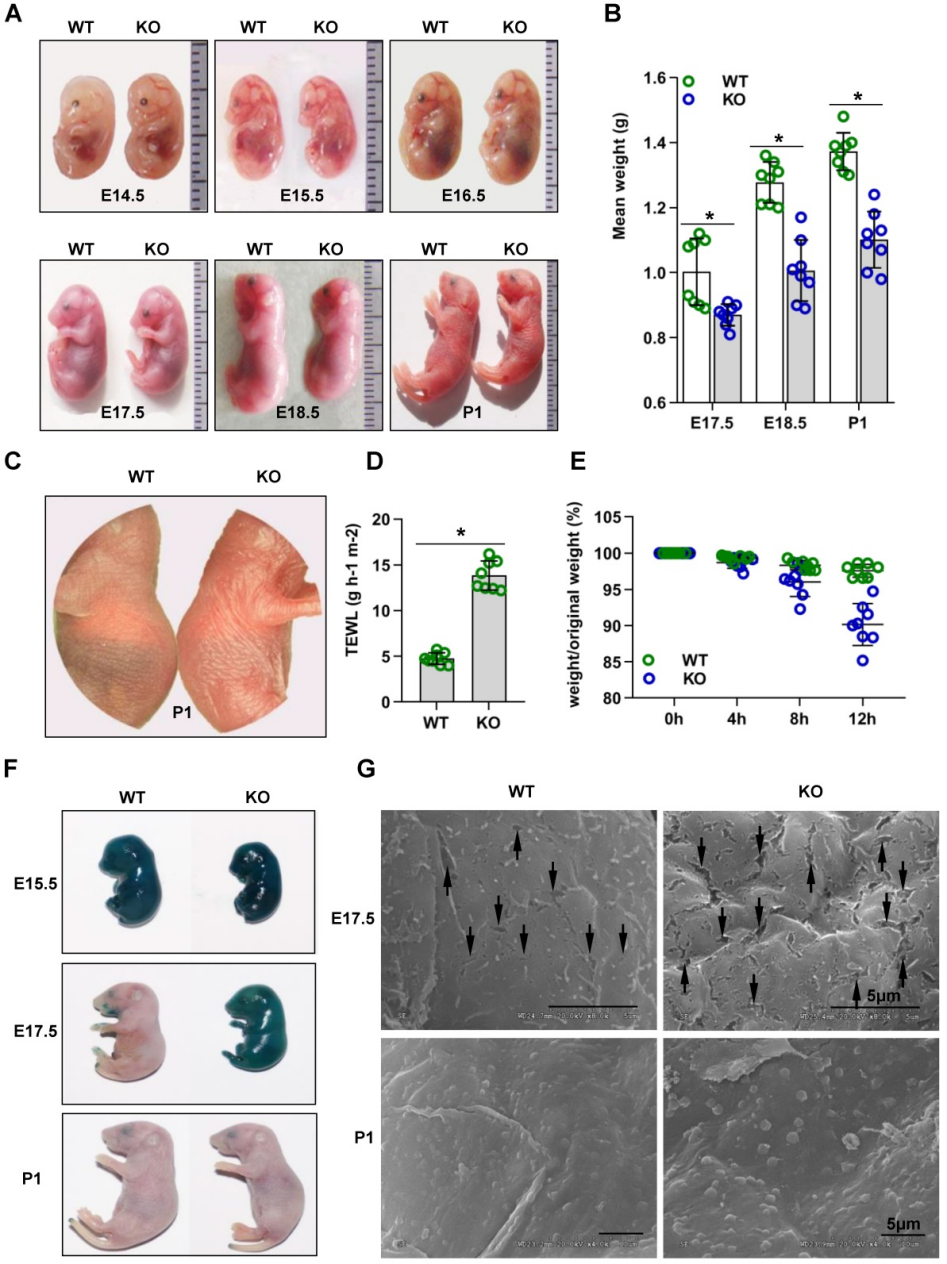
** Cdc42 knockout mice show rapid postnatal lethality and abnormal inside-out and outside-in barrier function.** (**A**) Cdc42 KO and WT control mice at E14.5, E15.5, E16.5, E17.5, E18.5, and P1. (**B**) The mean weight (g) of Cdc42 KO mice compared with that of WT littermates at E17.5, E18.5 and P1 (n = 8). (**C**) Comparison of the gross morphology of the skin in control and Cdc42 KO mice. (**D**) TEWL measurement at P1 (n = 8), *p<0.05. (**E**) The weight/birth weight ratios of Cdc42 KO and WT littermates at different time points (n = 8). (**F**) Skin permeability assay using X-gal staining in control and Cdc42 KO mice. (**G**) SEM of dorsal skin of E17.5 and P1 mice (the arrow shows a gap between epidermal corneocytes; n = 8). Scale bars: 5 μm.

Cdc42 KO mice were significantly smaller than Cdc42 heterozygous mice or WT littermate controls from E17.5 to P1 (**Figure 1A-B**). The lung, liver, kidney and heart of Cdc42 KO mice from E17.5 to P1 had no significant histological abnormalities **(Figure S2)**. The most important difference was that Cdc42 KO mice died within 24 h after birth.

### Cdc42 deletion impairs outside-in and inside-out barrier function during epidermal development

The skin of Cdc42 KO newborn mice exhibited a bright flush and tumefaction **(Figure 1C)**. Therefore, we next evaluated the outside-in and inside-out barrier function of the Cdc42 KO mouse epidermis. TEWL reflects inside-out barrier function 3. We found a considerable increase in TEWL in the P1 Cdc42 KO mice compared to the WT mice **(Figure 1D)**. Furthermore, Cdc42 KO mice lost approximately 5% of their body weight during their short lifespan, in comparison to an average of 1.5% loss in the control mice over the same period **(Figure 1E)**. Thus, the lethal phenotype of the Cdc42 KO mice most likely results from water loss due to a disturbance in epidermal barrier function.

In the X-gal staining assay 3, the dye penetrated the skin of both WT and KO mice at E15.5. However, while blue precipitate was absent from E17.5 and P1 WT embryos, it was still evident in E17.5 Cdc42 KO mice, not disappearing until P1 **(Figure 1F)**. These data suggest that the outside-in barrier was already formed by E17.5 in WT mice but not in Cdc42 KO mice. In support of this interpretation, SEM imaging showed larger gaps between epidermal corneocytes in E17.5 KO embryos than in their WT counterparts (**Figure 1G**). Together, our results suggest that Cdc42 deletion caused outside-in and inside-out barrier impairment during epidermal development.

### Cdc42 deletion causes loss of epidermal stem cells

**Figure 2:**
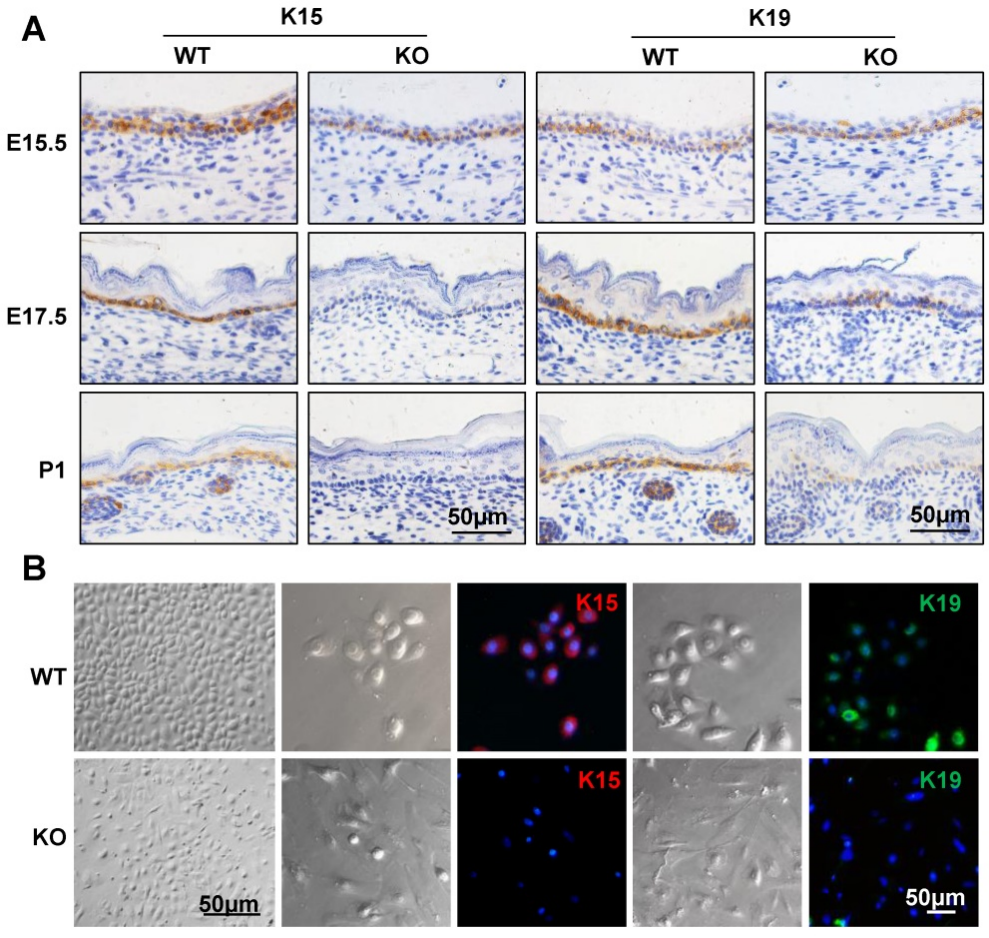
** Mice lacking Cdc42 showed loss of epidermal stem cells.** (**A**) K15 and K19 were detected by immunohistochemical staining in the basal layer of Cdc42 KO and WT mice at E15.5, E17.5, and P1. (**B**) Mouse primary keratinocytes were isolated from P1 Cdc42 KO and WT mice, cultured in low-calcium conditions (0.09 mM), and incubated with K15 or K19 antibodies. Scale bars: 50 μm.

To examine whether the epidermal stem cells were affected in the Cdc42 KO epidermis, immunohistochemical analysis was carried out to detect the expression of keratin 15 (K15) and keratin 19 (K19), which are markers of basal stem cells 28, 29. As shown in **Figure 2A**, there were few K15-positive cells in the basal layer of the Cdc42 KO mice epidermis at E17.5 and P1. K19-positive cells were detected in their usual location among the stem cells in the basal layer, but fewer K19-positive cells were detected in Cdc42 KO mice than in the controls** (Figure 2A)**. While primary keratinocytes from WT epidermis resembled epithelial cells in their cobblestone-like shape, keratinocytes from the Cdc42^-/-^ epidermis appeared to be fibroblast-like cells. There was significantly reduced expression of K15 and K19 in primary cultured epidermal stem cells from Cdc42 KO mice compared with controls. Moreover, Cdc42 KO stem cells failed to form colonies **(Figure 2B)**. These data confirm that Cdc42 deletion led to depletion of the stem cell compartment in the epidermis.

### Mice lacking Cdc42 show abnormal epidermal proliferation and apoptosis in epidermal development

**Figure 3:**
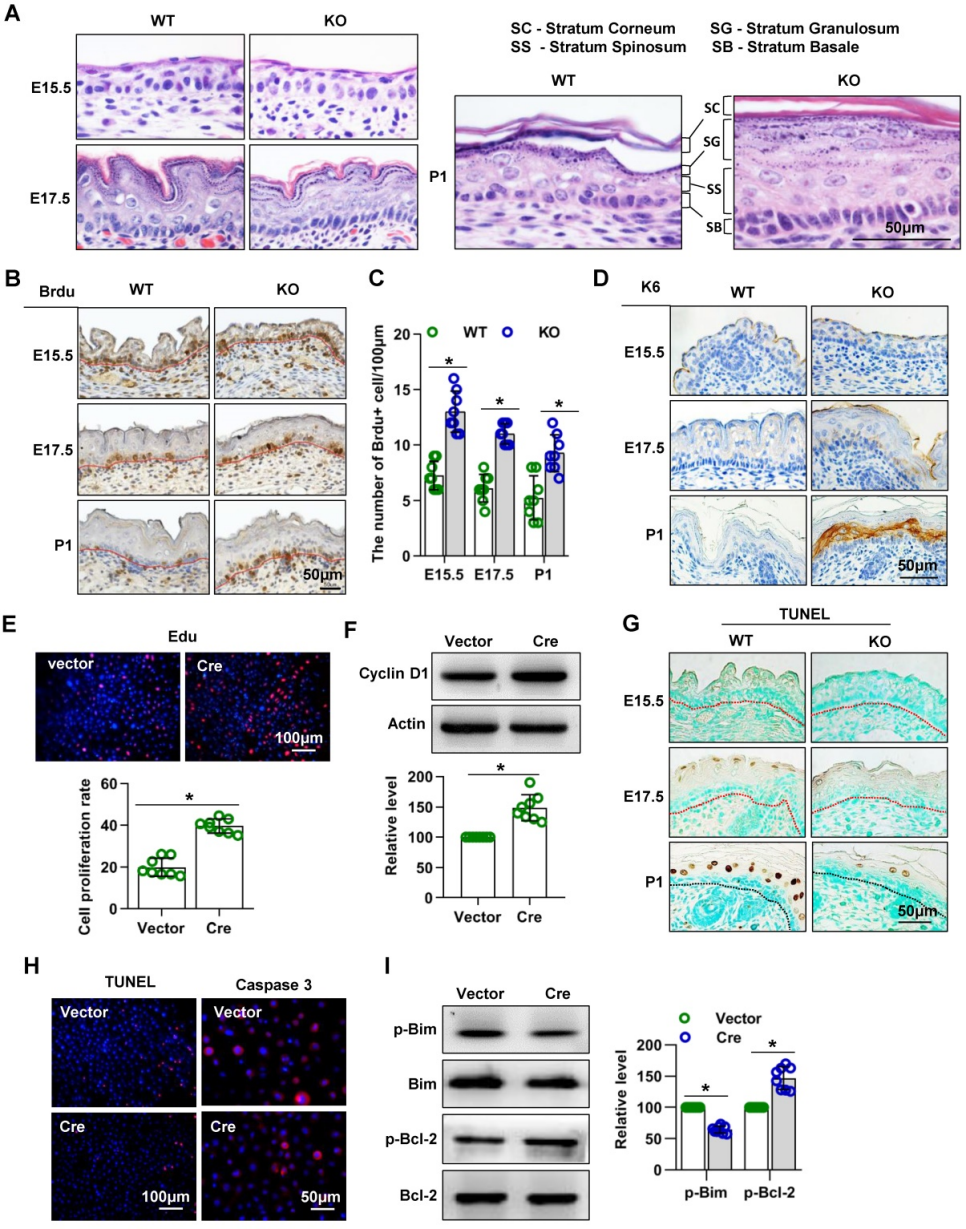
** Mice lacking Cdc42 show abnormal proliferation and apoptosis.** (**A**) H&E staining of control and Cdc42 KO mice at E15.5, E17.5 and P1. (**B**) Immunohistochemical detection of BrdU (E15.5-E18.5)-positive cells in the epithelium of Cdc42 KO and WT mice. (**C**) The number of BrdU-positive basal cells per 100 μm in the epidermis from eight independent experiments, n = 8. *p<0.05. (**D**) Immunohistochemical detection of K6 in the epidermis of E15.5 and P1 Cdc42 KO and WT mice. (**E**) Primary keratinocytes were isolated from P1 Cdc42^loxp/loxp/Cre-^ mice, cultured in low-calcium conditions, and then infected with vector or plenti-Cre. After 3 days, the proliferation of primary keratinocytes was detected using a Cell-Light EdU Apollo 567 In Vitro Kit. The number of EdU-positive cells per 200X field of view in eight independent experiments, n = 8. *p<0.05. (**F**) Detection of cyclin D1 by WB after primary keratinocytes were infected with vector or plenti-Cre; quantification of relative cyclin D1 from eight independent experiments. (**G**) Cell apoptosis detected by TUNEL staining. TUNEL-positive cells appear yellowish brown, and the cell nuclei appear blue (n = 8). (**H**) The cells were treated the same way as in **(E)**, and apoptosis was detected using a TUNEL Bright Red Apoptosis Detection Kit. Caspase 3 was detected by immunofluorescence. The TUNEL- positive and caspase 3-positive cells were counted at 200X and 400X magnification, respectively, and the quantification was based on eight independent experiments. (**I**) Bcl-2, p-Bcl-2, Bim, and p-Bim detected by WB after primary keratinocytes were infected with vector or plenti-Cre. The quantification was based on eight independent experiments.

H&E staining of dorsal skin sections revealed severe alterations of the epidermis in the absence of Cdc42: The epidermis of Cdc42 KO mice, especially the SS and SC, was thicker than that of WT mice at E17.5 and P1. The nuclei of basal cells of the interfollicular epidermis had a more elongated shape in Cdc42 KO mice than in control mice at E17.5 and P1; this feature is often observed in hyperproliferative epidermis **(Figure 3A).**

We next investigated whether the epidermal thickening in Cdc42 KO mice was due to increased cell proliferation. We detected more BrdU-positive cells in the epidermal basal layer of Cdc42^-/-^ mice than in that of WT controls at each stage from E15.5 to P1 **(Figure 3B-C).** Furthermore, expression of K6, a hyperproliferation-related keratin 30, was absent from the epidermis of WT controls but gradually increased in the SS of the epidermis across E17.5 and P1 in Cdc42 KO mice (**Figure 3D**).

Keratinocytes isolated from Cdc42 KO epidermis in P1 failed to form colonies, as shown in Figure 2B. Therefore, in order to investigate the role of Cdc42 in keratinocyte proliferation, keratinocytes were isolated from Cdc42 flox/flox animals and transfected with the Cre lentivirus. The transfection efficiency was assessed by GFP imaging and WB (**Figure S3A-B**). We found that deletion of Cdc42 caused excessive proliferation of keratinocytes **(Figure 3E)**, which was associated with the upregulation of cyclin D1 **(Figure 3F)**.

Additionally, we investigated the role of Cdc42 in apoptosis. There were fewer TUNEL^+^ cells in the epidermis of Cdc42 KO mice than in that of WT control mice at P1 **(Figure 3G)**. In keratinocytes, the TUNEL assay also showed that the loss of Cdc42 inhibited apoptosis, while the expression of caspase 3, a protease that is important in apoptosis 31, was also reduced **(Figure 3H)**. The WB results showed that the phosphorylation level of Bim, a proapoptotic protein 32, was decreased, whereas that the phosphorylation of Bcl-2, an antiapoptotic protein 32, was increased **(Figure 3I)**.

Taken together, these data showed that Cdc42 deletion promoted keratinocyte proliferation and inhibited cell apoptosis, which likely accounted for the thickening of the epidermis.

### Cdc42 deficiency results in abnormal TJs, adherens junctions and desmosomes in the epidermis during its development

**Figure 4:**
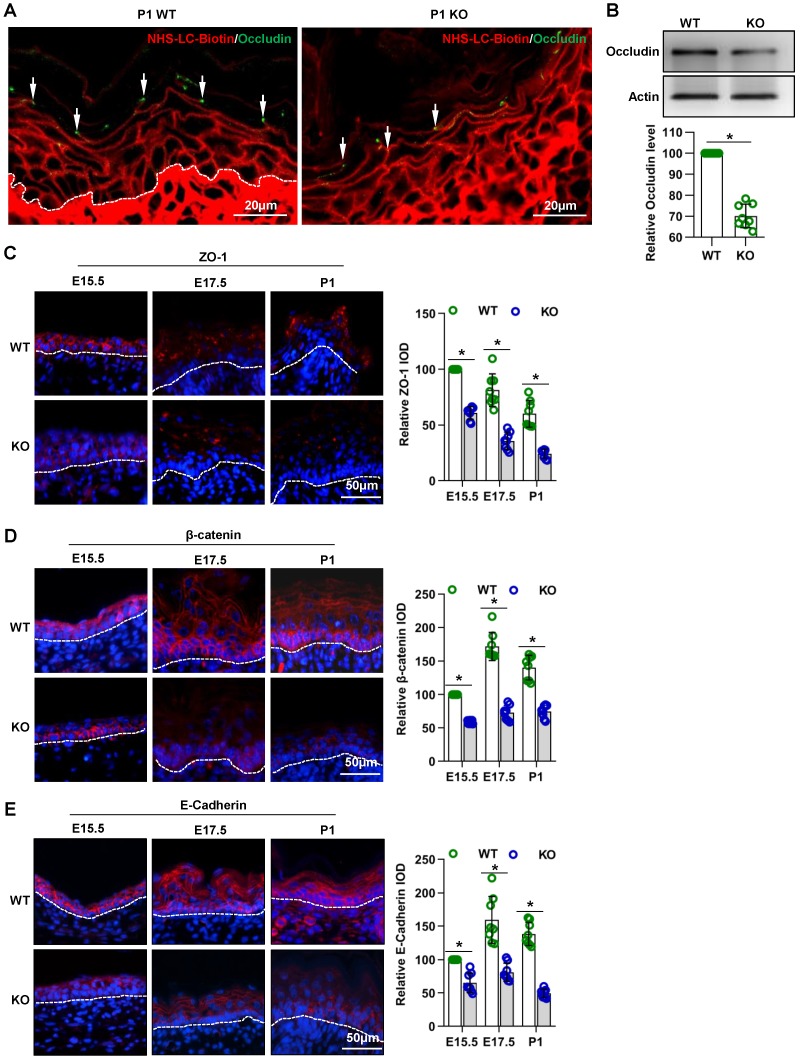
** Cdc42 deficiency results in abnormal TJs and adherens junctions in the epidermis.** (A) EZ-Link™ Sulfo-NHS-LC-Biotin was injected into the dermis on the backs of Cdc42 KO and WT neonates, and after 30 min of incubation, the skin was dissected away and frozen. The frozen sections were double stained with streptavidin-conjugated anti-occludin (green, to label TJs) and with biotin (red). In the P1 WT epidermis, the NHS-LC-biotinylation reagent diffused through the paracellular spaces from the SB to the SG, but this diffusion was abruptly stopped at occludin-positive TJs (arrows). In the P1 KO epidermis, diffusion was not prevented at most occludin-positive TJs. Instead, the NHS-LC-biotinylation reagent passed through those TJs to reach the border between the SG and the SC. **(B)** The epidermis was isolated from P1 WT and KO mice, and the expression of occludin was detected by WB; the quantification was based on eight independent experiments. Expression, distribution, and immunofluorescence semiquantification of ZO-1 **(C)**, β-catenin** (D)**, and E-cadherin** (E)** in Cdc42 KO and control mice. n = 8, *p<0.05.

Intercellular junctions are associated with the “inside-out” barrier function of the skin 3. The cell- cell junctions in skin epidermis includes the tight junction, adherens junction, and desmosomes 33. In this study, the TJ permeability assay showed that the NHS-LC-biotinylation reagent diffused through the paracellular spaces from the SB to the SG in the P1 WT epidermis, but this diffusion was abruptly stopped at TJs staining positive for occludin **(Figure 4A, arrows)**, an important TJ protein 20. However, in the P1 KO epidermis, diffusion was not prevented at most occludin-positive TJs. Instead, the NHS-LC-biotinylation reagent passed through these TJs and reached the border between the SG and the SC. Moreover, the expression of occludin was decreased in the epidermis of mutant mice** (Figure 4A-B)**.

The immunohistochemistry (**Figure 4C-E**) and qPCR (**Figure S4A-C**) results showed that the expression of the TJ marker ZO-1 and of the adherens junction proteins E-cadherin and β-catenin 34, 35 were decreased in the epidermis of mutant mice.

**Figure 5:**
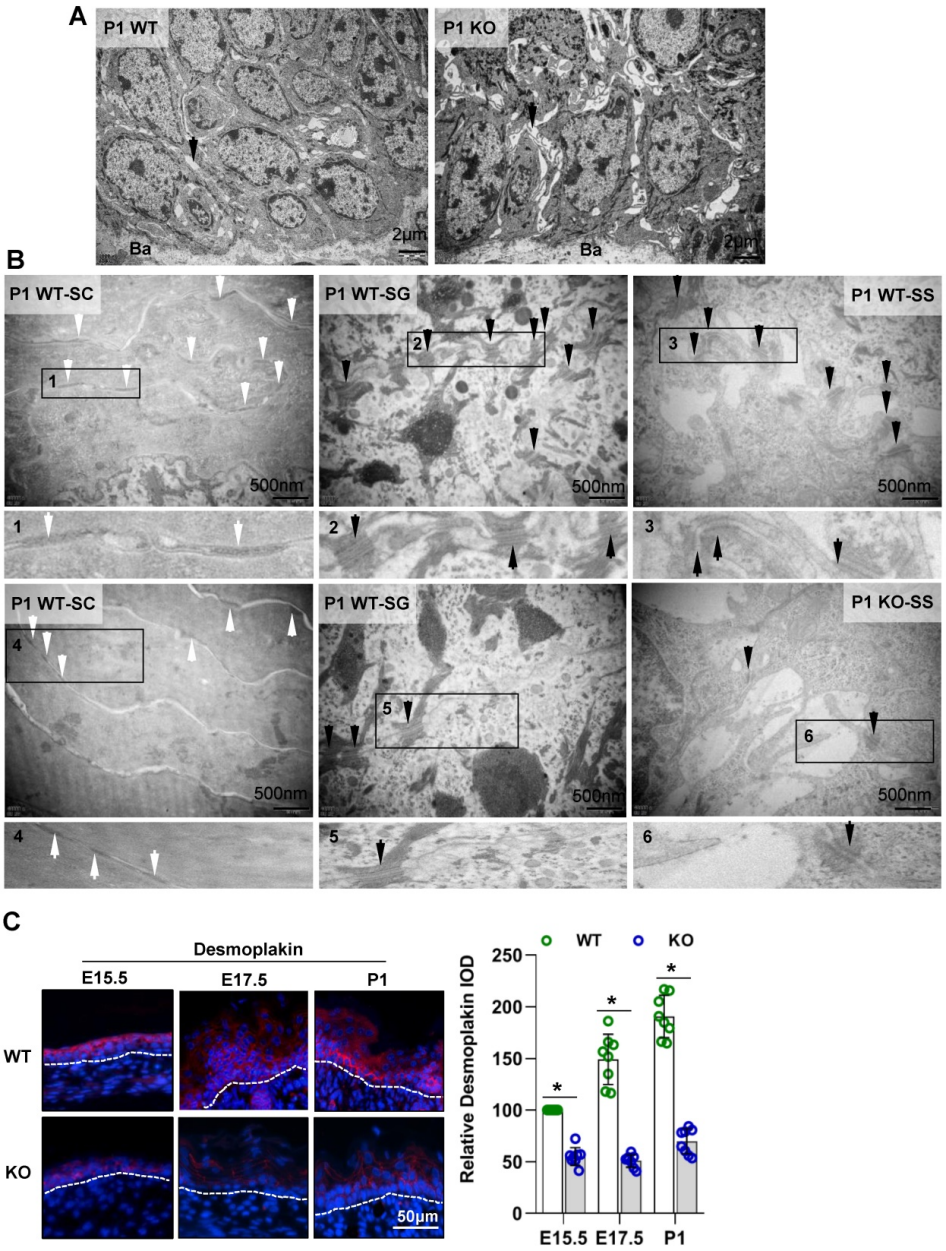
** Cdc42 deficiency results in abnormal desmosomes in the epidermis. (A**) TEM of the full-thickness dorsal skin of P1 mice. Black arrows indicate intervals between cells in the basal and spinous layer. n = 8 mice per genotype. Ba, basilemma. Scale bars: 2 μm. **(B)** TEM of the SC, SG and SS of P1 mice. White arrows indicate corneodesmosomes in the SC, and black arrows indicate desmosomes in the SG and SS. **(C)** Expression and distribution and IF semiquantification of desmoplakin in Cdc42 KO and control mice. n = 8, *p<0.05.

In addition, TEM imaging showed that the cell interval (**arrows in Figure 5A**) was increased. The numbers of corneodesmosomes (**white arrows in Figure 5B**) 2 in the SC and desmosomes (**black arrows in Figure 5B**) in the SG and SS were also reduced in the Cdc42 KO epidermis. The immunohistochemistry (**Figure 5C**) and qPCR (**Figure S4D**) results showed that the desmosome marker desmoplakin 36 was reduced in the Cdc42 KO epidermis compared to that of the WT control. These results suggest that loss of Cdc42 caused defects in intercellular connections.

### Cdc42 affects the expression of E-cadherin and ZO-1 through PAK1

**Figure 6:**
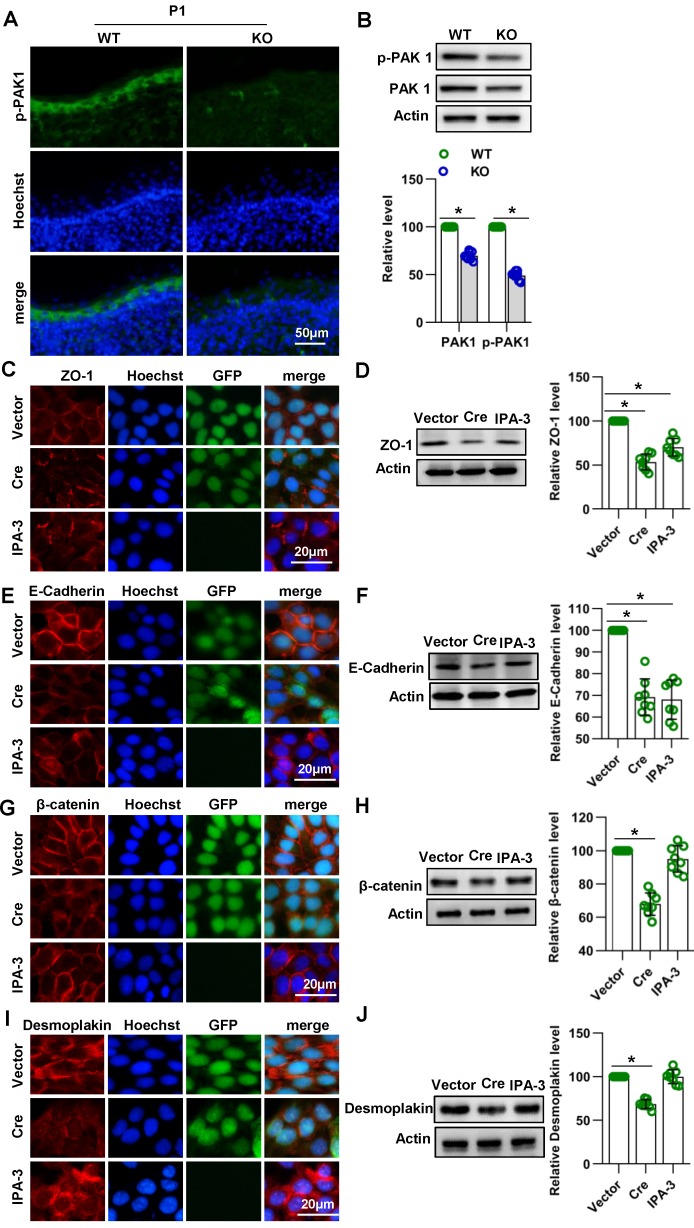
** Cdc42 affect the expression of E-cadherin and ZO-1 through PAK1.** (**A**) IF detection of PAK1 in the epidermis of KO and WT mice at E15.5, E17.5 and P1 Cdc42. (**B**) The epidermis was isolated from P1 WT and KO mice, and the expression of PAK1 and p-PAK1 was detected by WB; the quantification was based on eight independent experiments. Primary keratinocytes were isolated from P1 Cdc42^loxp/loxp/Cre-^ mice and then infected with vector or plenti-Cre, grown to confluency in low-calcium medium and then incubated in high-calcium medium for 12 h. In the IPA-3 group, keratinocytes were incubated for 12 h in high-calcium medium with the PAK1 inhibitor IPA-3 (5 μm, Selleck Chemicals). Cells were stained for ZO-1 (**C**), β-catenin (**E**), E-cadherin (**G**) and desmoplakin (**I**). Vector-infected keratinocytes, plenti-Cre-infected keratinocytes and IPA-3 keratinocytes were grown in low-calcium medium, and WB of cell lysates was performed with antibodies against ZO-1 (**D**), β-catenin (**F**), E-cadherin (**H**) and desmoplakin (**J**). Actin was used as a loading control. The experiments were repeated at least eight times; *p<0.05.

In epithelial cells, PAK1 has been found to regulate E-cadherin endocytosis and ZO-1 localization and maturation in epithelial cells 37. PAK1 is a serine/threonine kinase and an important downstream signaling molecule of Cdc42 37, 38. To investigate whether Cdc42 affects junctions between keratinocytes by acting through PAK1, we evaluated the expression and activity of PAK1 in Cdc42 knockout epidermis. Immunofluorescence and WB studies showed that the expression and activity of PAK1 were downregulated in Cdc42-deleted epidermis (**Figure 6A-B**). Furthermore, by the use of PAK1 inhibitor IPA-3, we found that PAK1 inhibition affected the expression of E-cadherin and ZO-1 in keratinocytes (**Figure 6C-6F**), but its effects on desmoplakin and β-catenin were not significant (**Figure 6G-6J**). These results suggest that Cdc42 may affect the expression of E-cadherin and ZO-1 through PAK1.

### Mice lacking Cdc42 show abnormal epidermal differentiation and overexpression of several SPRR family members in epidermal development

To investigate the effects of Cdc42 deletion on epidermal homeostasis and differentiation, we examined the expression of the basal cell marker K14 and the differentiation marker K1 by immunofluorescence. While K14 expression was confined to the basal layer of the epidermis in E15.5-P1 WT mice, its expression expanded to the SS and the SG of the epidermis in E17.5-P1 Cdc42 KO mice. In addition, the absence of Cdc42 led to increased expression of K1 **(Figure S5A-B)**.

**Figure 7:**
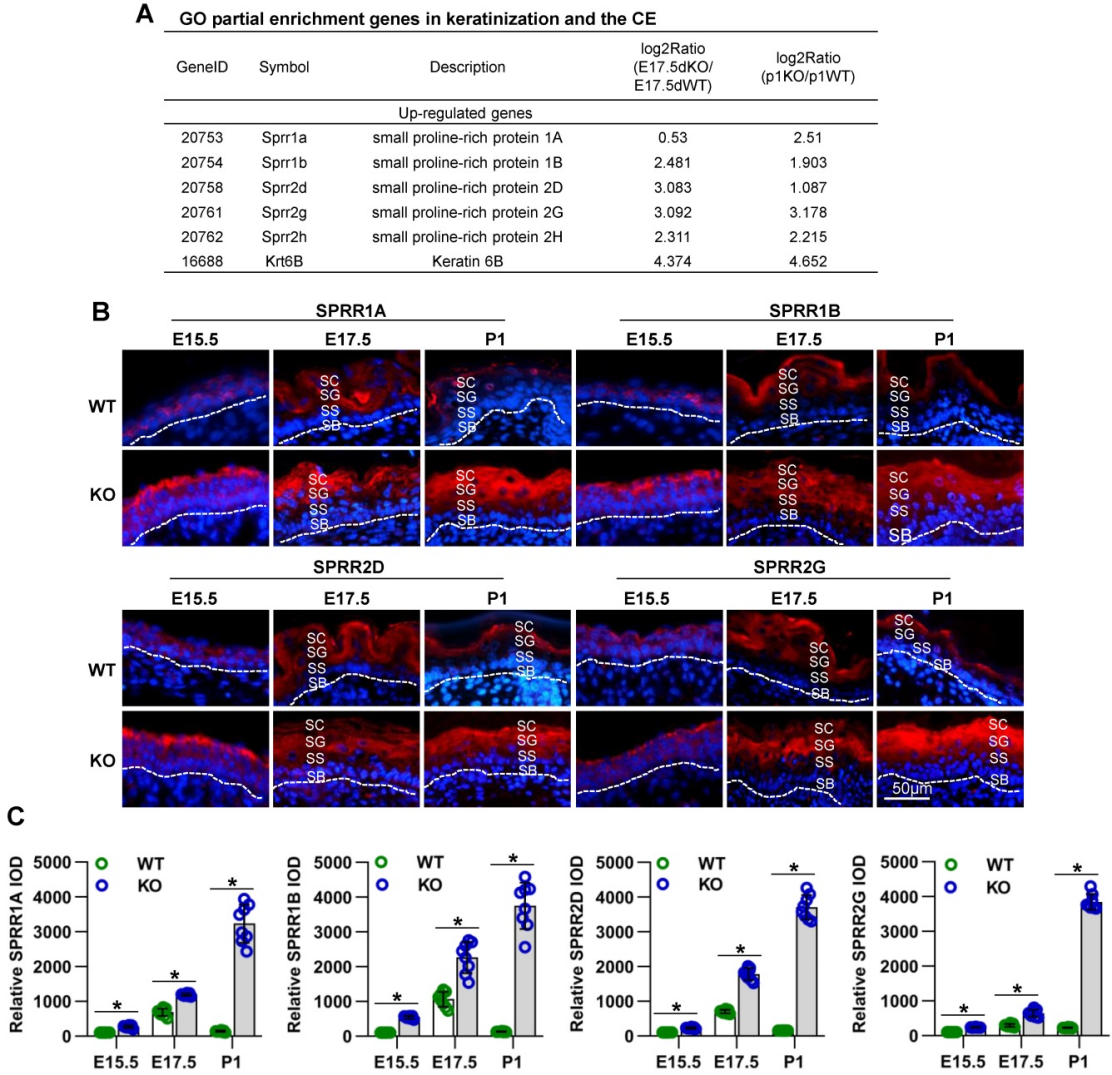
** Cdc42 deficiency results in the overexpression of several SPRR family members.** (**A**) Common DEGs in the CE (SPRR1A, SPRR1B, SPRR2D, SPRR2G, SPRR2H, and KRT6B). (**B**) Expression and distribution of SPRR1A, SPRR1B, SPRR2D and SPRR2G in Cdc42 KO and control mice (n = 8). Scale bars: 50 μm. (**C**) Immunofluorescent semiquantification of SPRR1A, SPRR1B, SPRR2D and SPRR2G in the Cdc42 KO epidermis (n = 8); *p<0.05.

To explore the molecular mechanisms of Cdc42 in skin development, we analyzed skin from WT and Cdc42^-/-^ mice at E17.5 and P1 by RNA sequencing. We found that the most significantly altered genes in the E17.5 and P1 mutant skins were enriched in the GO terms “keratinization” and “cornified envelope” **(Figure S6A-B)**, both related to the physical structure of the epidermal barrier 9. Among the DEGs in the CE term, several members of the SPRR family were upregulated in Cdc42^-/-^ skin (**Figure 7A**). We confirmed the upregulation of SPRR1A, SPRR1B, SPRR2D, SPRR2H, and SPRR2G by qPCR** (Figure S4E)**.

Although SPRR family members are well known to be important members of the cornified envelope, with potential functions in barrier formation 9, the roles of these proteins in epidermal development remain unclear. Immunohistochemical analysis revealed that the expression of SPRRs during epidermal development is spatiotemporally dependent. The locations of SPRR1A, SPRR1B, SPRR2D, and SPRR2G expression in WT mice underwent dynamic changes accompanied by the differentiation and keratinization of epidermal cells. At E14.5, those four proteins were expressed in all layers in the developing epidermis; at E15.5, they were expressed in the SC, SG and SS; at E17.5, they were expressed in the SC and SG; at P1, they were expressed only in the SC. However, the lack of Cdc42 in the basal layer not only led to increased expression of SPRR family members but also changed the location of their expression at E15.5 to P1, during which time were continuously expressed in all layers above the SB **(Figure 7B-C)**. However, we found that the expression of other CE-related proteins, such as involucrin and loricrin, was not affected in Cdc42 KO mice (**Figure S5B**).

These data suggest that SPRR family members may have a function in epidermal barrier dysfunction induced by Cdc42 deletion.

### SPRR1B overexpression led to abnormal cell proliferation and cell-cell junctions in the epidermis

Although some studies have shown that SPRR family member's expression is elevated in psoriatic plaques and atopic dermatitis, conditions that are associated with skin barrier abnormalities 39-41, the role of this family member's overexpression in the epidermis needs to be clarified. As SPRR1 gene induction plays an important role in the oral epithelial barrier formation, we firstly investigated the role of SPRR1B, which is overexpression in Cdc42 KO epidermis both at E17.5 and E19.5, in the epidermis.

**Figure 8:**
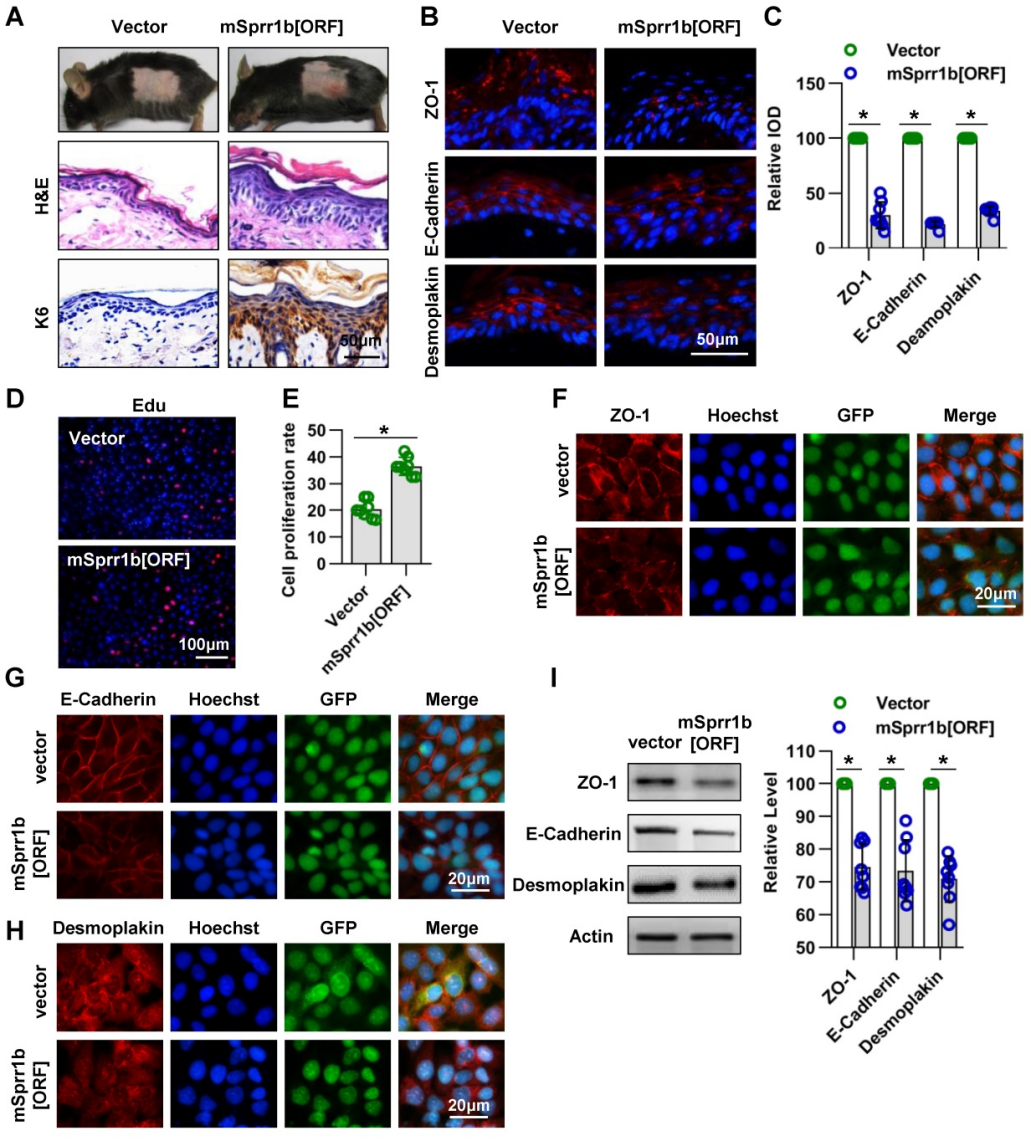
** Overexpression of SPRR family members cause abnormal cell junctions. (A**) The lentivirus vectors and mSprr1b[ORF045957] were applied to mouse skin. After 3 days, H&E staining was performed. The expression and distribution of K6 were determined by immunohistochemistry. (**B**) The expression and distribution of ZO-1, E-cadherin and desmoplakin were determined semiquantitatively by immunofluorescence (**C**); n = 8; *p<0.05. (**D**) Primary keratinocytes were isolated from P1 WT mice, cultured in low-calcium conditions, and then infected with vector or msprr1b[ORF]. After 3 days, the proliferation of primary keratinocytes was detected using a Cell-Light EdU Apollo 567 In Vitro Kit. (**E**) The number of EdU-positive cells per 200X field of view from eight independent experiments, n = 8; *p<0.05. Primary keratinocytes were infected with vector or msprr1b[ORF], grown to confluency in low-calcium medium and then incubated in high-calcium medium for 12 h. Cells were stained for ZO-1 (**F**), E-cadherin (**G**) and desmoplakin (**H**). (**I**) Vector-infected keratinocytes and msprr1b[ORF]-infected keratinocytes were grown in low-calcium medium, and WB of cell lysates was performed with anti-ZO-1, E-cadherin and desmoplakin antibodies. Actin was used as a loading control. The experiments were repeated at least eight times; *p<0.05.

Lentivirus expressing mutant mSprr1b [ORF] was applied to the dorsal skin of 7-week-old C57BL/6 mice. The overexpression of SPRR1B in the epidermis was confirmed by WB three days after lentiviral infection** (Figure S3C-D)**. Histological examination revealed that SPRR1B overexpression led to thickening of the skin, especially the SS **(Figure 8A)**. Immunohistochemistry analysis showed that K6 was overexpressed in SPRR1B-overexpressing epidermis **(Figure 8A)**, suggesting hyperproliferation of epidermal cells. Moreover, using immunohistochemistry, we found, to our surprise, that the expressions of ZO-1, E-cadherin and desmoplakin were decreased upon SPRR1B overexpression (**Figure 8B-C**).

Furthermore, the roles of SPRR1B in keratinocyte proliferation and cell-cell junctions were confirmed *in vitro*. The transfection efficiency was confirmed by WB (**Figure S3E-F**), and SPRR1B overexpression was found to lead to the excessive proliferation of keratinocytes** (Figure 8D-E)**. In addition, the expression levels of ZO-1, E-cadherin and desmoplakin were decreased when SPRR1B was overexpressed in keratinocytes **(Figure 8F-I)**.

Taken together, these data illustrate that SPRR1B overexpression caused keratinocyte hyperplasia and dampened cell junction proteins expression in epidermis.

### Overexpression of SPRR1B or SPRR2D is partially responsible for cell-cell junctions and skin barrier dysfunction resulting from Cdc42 suppression

Next, we intended to reverse SPRR1B expression in the Cdc42^-/-^ epidermis to investigate whether the upregulation of SPRR1B and its fellow SPRR family member SPRR2D was responsible for the skin barrier dysfunction and abnormal intercellular junctions of Cdc42-deficient mice. However, it is technically challenging to reverse SPRR1B expression in the Cdc42^-/-^ epidermis during embryonic development or in moribund P1 mice. Therefore, we applied a virus expressing a dominant negative Cdc42 mutant (Cdc42N17) to inhibit Cdc42 activity in the epidermis of adult mice. EGFP expression indicated efficient expression of Cdc42N17, and Cdc42 activity (Cdc42-GTP) was inhibited in the epidermis **(Figure S3G-H)**. We found that the outside-in skin barrier was damaged after infection with Cdc42N17 **(Figure S7A).** As in the Cdc42 KO mice, a considerable increase in TEWL was found after Cdc42N17 infection **(Figure S7B).** Similar to the Cdc42 KO epidermis, the Cdc42- suppressed epidermis showed increased expression of SPRR1B, SPRR2D, and SPRR2G that expanded from the SC to all other layers except the SB **(Figure S7C-D)**.

**Figure 9:**
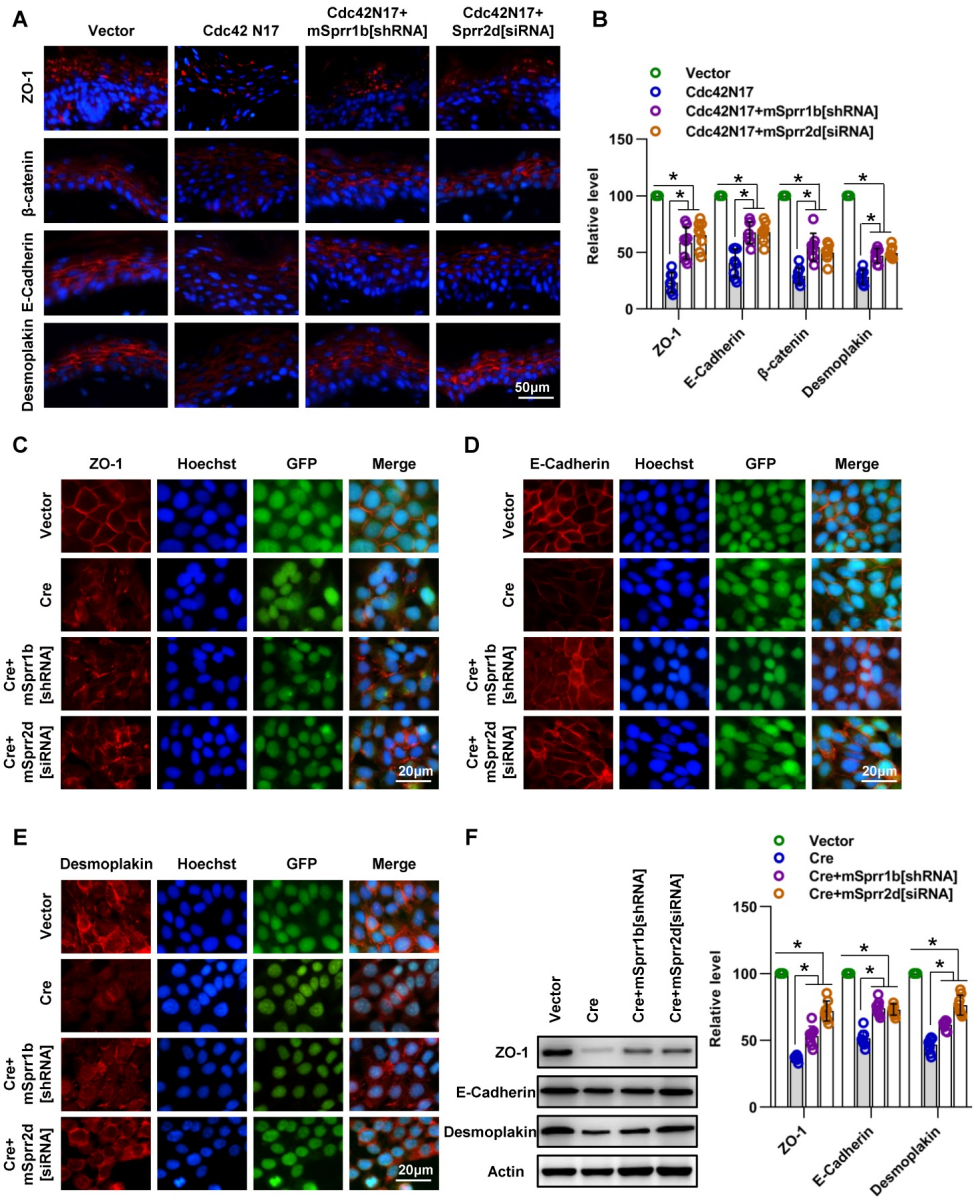
** SPRR1B and SPRR2D are partially responsible for altered intercellular junctions and skin barrier dysfunction resulting from Cdc42 suppression. (A**) The lentiviral vectors Cdc42N17 were applied to the skin of C57BL/6 mice. The mouse skin was transfected with Cdc42N17 and mSprr1b[shRNA] vectors at the same time. For the Cdc42N17+mSprr2d[siRNA] group, the skin was transfected with mSprr2d[siRNA] for 3 days and then infected with Cdc42N17 vectors for another 3 days. The expression and distribution of ZO-1, β-catenin, E-cadherin and desmoplakin were determined by immunohistochemistry.** (B)** Immunofluorescent semiquantification of ZO-1, β-catenin, E-cadherin and desmoplakin. n = 8, *p<0.05. Primary keratinocytes were isolated from P1 Cdc42^loxp/loxp/Cre-^ mice and then infected with vector or plenti-Cre; in the plenti-Cre+mSprr1b[shRNA] group, keratinocytes were infected with plenti-Cre and mSprr1b[shRNA] vectors; in the plenti-Cre+ mSprr2d[siRNA] group, keratinocytes were infected with plenti-Cre and then treated with mSprr2d[siRNA]. The cells were grown to confluence in low-calcium medium for 24 hours and then incubated in high-calcium medium for 12 h. Cells were stained for ZO-1 (**C**), E-cadherin (**D**) and desmoplakin (**E**). (**F**) Vector-infected keratinocytes, plenti-Cre-infected keratinocytes, plenti-Cre+ mSprr1b[shRNA] keratinocytes, and plenti-Cre+ mSprr2d[siRNA] keratinocytes were grown in low-calcium medium, and WB of cell lysates was performed with anti-ZO-1, E-cadherin and desmoplakin antibodies. Actin was used as a loading control. The experiments were repeated at least eight times; *p<0.05.

We then examined whether downregulation/ reversal of SPRRs in Cdc42-suppressed skin induced intercellular junction protein and TEWL recovery. We found that SPRR1B shRNA and Sprr2d[siRNA] partially rescued the defect in cell-cell junction integrity and TEWL induced by Cdc42N17 **(Figure 9A-B and Figure S7E)**. Furthermore, we confirmed that SPRR1B[shRNA] and Sprr2d[siRNA] partially rescued the defect in cell-cell junction integrity induced by Cdc42 knockout in keratinocytes *in vitro* **(Figure 9C-F)**.

These data suggest that overexpression of SPRR1B or SPRR2D is partially responsible for cell- cell junctions and skin barrier dysfunction resulting from Cdc42 suppression.

### Cdc42 regulates the expression of SPRR1 through the MAPK-AP-1 signaling pathway

**Figure 10:**
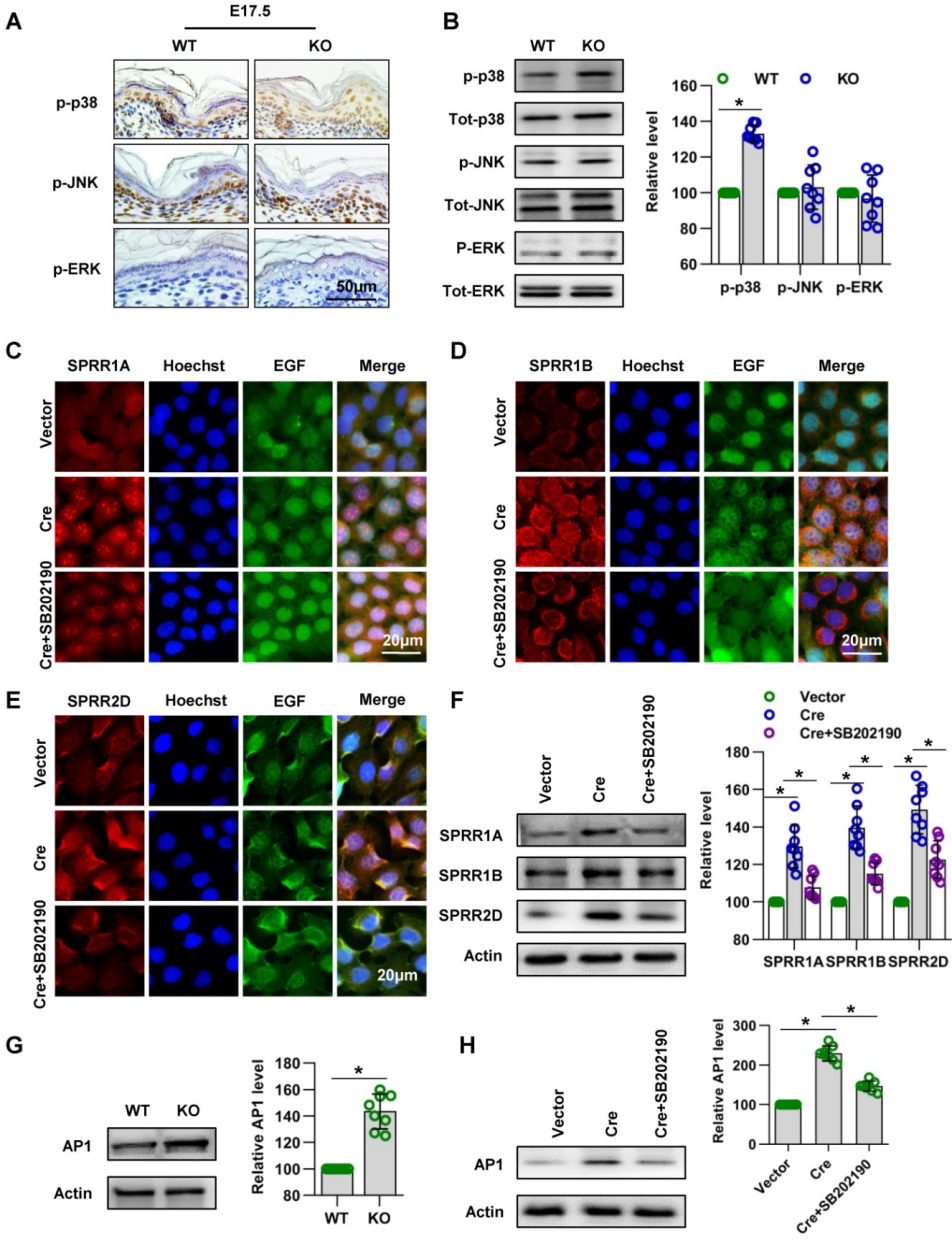
** Cdc42 regulates the expression of the SPRR family through the MAPK1-AP-1 signaling pathway. (A**) Immunohistochemical detection of p-p38, p-JNK, and p-ERK in the epidermis of P1 Cdc42 KO and WT mice. (**B**) The epidermis was isolated from P1 WT and KO mice, and the expression of p-p38, p38, p-JNK, JNK, p-ERK and ERK was detected by WB; the quantification was based on eight independent experiments. Primary keratinocytes were isolated from P1 Cdc42^loxp/loxp/Cre-^ mice and then infected with vector or plenti-Cre, cultured in low-calcium medium for 3 days and then incubated in high-calcium medium for 24 h. In the SB202190 group, keratinocytes were incubated for 24 h in high-calcium medium with the p38 inhibitor SB202190 (5 mM, Selleck Chemicals). Cells were stained for SPRR1A (**C**), SPRR1B (**D**) and SPRR2D (**E**). (**F**) Vector-treated keratinocytes, plenti-Cre-treated keratinocytes and SB202190 keratinocytes were incubated for 24 h in high-calcium medium, and WB of cell lysates was performed with anti-SPRR1A, SPRR1B and SPRR2D antibodies. Actin blots were used as loading controls. The experiments were repeated at least eight times; *p<0.05. (**G**) The epidermis was isolated from P1 WT and KO mice, and the expression of AP-1 was detected by WB; the quantification was based on eight independent experiments. *p<0.05. (**H**) As in (G), WB of cell lysates was performed with anti-AP-1 antibody. Actin was used as a loading control. The experiments were repeated at least eight times; *p<0.05.

Subsequently, we explored the signaling mechanism by which Cdc42 regulates the expression of SPRR family. A series of studies suggested that MAPK signaling pathways play an important role in the expression of the SPRR family 42-44. In order to investigate the signaling mechanism in the upregulation of SPRR expression induced by Cdc42 deletion, three different MAPK signaling pathways, the MAPK/JNK, MAPK/ERK and p38 MAPK pathways, were first examined. Immunohistochemistry and WB showed that the expression and activity of p38 were upregulated in Cdc42-deleted epidermis **(Figure 10A-B).** Furthermore, when the activity of p38 was inhibited by its specific inhibitor SB202190, the expression levels of SPRR1A and SPRR1B were decreased, but the expression of SPRR2D was not significantly altered in keratinocytes (**Figure 10C-F**). These results suggested that Cdc42 may affect the expression of SPRR1 through p38 MAPK.

AP-1 is the key transcription factor regulating SPRR transcription and expression 45-47. In this study, we found that Cdc42 knockout induced the upregulation of AP-1 expression in P1 mice **(Figure 10G)**. Furthermore, this study explored the effect of MAPK on AP-1 activity and SPRR expression. We found that p38 inhibitors induce the downregulation of AP-1 expression in keratinocytes **(Figure 10H)**. The upregulation of SPRR mediated by Cdc42 knockout was due to the upregulation of the MAPK-dependent AP-1 signaling pathway.

## Discussion

### Cdc42 is an important regulator of epidermal outside-in and inside-out barrier acquisition in the fetus

During development, the fetus requires a barrier to prevent intrauterine infection and to serve as an interface with the amniotic fluid 41. In this study, we found that at E17.5, the skin outside-in barrier was well formed in WT mice but damaged in Cdc42 KO mice. Furthermore, most of the Cdc42 KO mice exhibited weight loss at the same time. Considering that the important organs (lung, liver, kidney, heart) of Cdc42 KO mice had no evident histological abnormalities at E17.5, we hypothesized that the destruction of the skin barrier of Cdc42 KO mice may result in intrauterine infection induced by disruption of the outside-in barrier. In support of this hypothesis, we found large numbers of inflammatory cells in the liver and subcutaneous tissue of E17.5 and P1 Cdc42 KO mice (**Figure S8A**).

In addition, we found that the inside-out barrier in Cdc42 KO mice was incomplete at both E17.5 and P1, which lead to water loss. Thus, the lethal phenotype of the Cdc42 KO mice was most likely due to infection and dehydration. Collectively, these results indicate that Cdc42 is an important regulator of the acquisition of the epidermal outside-in and inside-out barrier.

### Cdc42 in the regulation of epidermal stem cells and the balance between epidermal cell proliferation and differentiation in epidermal development

Epidermal development coordinates keratinocyte survival, proliferation, differentiation, and apoptosis. In this study, we demonstrated that Cdc42 is involved in the regulation of these processes.

We found that Cdc42 deletion resulted in loss of epidermal stem cells. Interestingly, deletion of Rac1, another Rho GTPase, also stimulated epidermal stem cells to divide and undergo terminal differentiation, which led to rapid depletion of stem cells and failure to maintain the interfollicular epidermis 48. One key mechanism by which Rac1 maintains epidermal stem cells is by negatively regulating Myc through PAK2 phosphorylation 48. Although the exact mechanism leading to stem cell loss in Cdc42 deleted epidermis remains to be determined, these findings suggest that both Cdc42 and Rac1 are required for the maintenance of epidermal stem cell hemostasis.

Growth and differentiation, a tightly linked pair of processes in epidermal development and homeostasis, need to be precisely balanced 49. In the present study, we demonstrated that Cdc42 deletion in the epidermis resulted in an imbalance between cell proliferation and differentiation in epidermal development. CyclinD1 is closely associated with the proliferation of the epidermis 50. The increase of this critical signal might, at least in part, account for the proliferation induce by Cdc42 deletion in mutants. Consistent with this finding, Wu's study also showed that Cdc42 deficiency resulted in strong hyperplasia and hyperkeratosis of the epidermis in adult mice 17. Taken together, the present study and Wu's study show that Cdc42 is a key regulator of the balance of epidermal proliferation and differentiation both in epidermal development and in adult epidermal homeostasis.

Furthermore, we found that Cdc42 deletion inhibited keratinocyte apoptosis, and was associated with reduced Caspase 3 expression and Bim activity and increased Bcl2 activity. Our data suggest that Cdc42 may be regarded as an anti-apoptosis protein in epidermal development. Consistent with our findings, recent studies have demonstrated that Cdc42 is an anti-apoptosis protein in podocytes 51.

### Cdc42 in the regulation of cell-cell junctions

The epidermis contributes to the inside-out barrier through intercellular junctions 12, 33. We showed that Cdc42 deficiency resulted in alterations in three types of intercellular junctions in the epidermis: TJs (indicated by the marker ZO-1), adherens junctions (indicated by the markers E-cadherin and β-catenin), and desmosomes (indicated by the protein desmoplakin). Considering that loss of E-cadherin, ZO-1 and desmoplakin alone can cause neonatal death and epidermal barrier damage 52-54, alterations in these proteins may have resulted in inside-out barrier defects in Cdc42 KO mice. In addition, our findings corroborate other studies, which have also revealed a critical role of Cdc42 in regulating the structure and function of epithelial cell-cell junctions. For example, an *in vitro* study by Dr. Du revealed that Cdc42, by regulating the activation of aPKCζ, was crucial for the formation of mature epithelial cell junctions between keratinocytes 25. In Madin-Darby canine kidney (MDCK) epithelial cells, Cdc42 exerts an effect on the epithelial barrier by regulating the TJ protein claudin-2 55.

Furthermore, our data indicated that PAK1 is involved in the regulation of disruption of E-cadherin and ZO-1 induced by Cdc42 deletion. The PAK family is an important regulator of cell adhesion, motility and survival. Our results suggest that PAK1 is a pivotal Cdc42 target that coordinates junction disassembly. In support of this suggestion, Mario and his colleagues found that Pak1 is the downstream effector of Cdc42 in the regulation of ZO-1 in human Caco2 intestinal cells 37.

Mice generated by K14-cre-mediated excision of the Cdc42 gene display a related but more severe phenotype than do mice that express Cre under the K5 promoter. Consistent with this observation, in previous work, the junctional proteins E-cadherin, β-catenin, and ZO-1 did not show evidence of disturbances in Cdc42fl/fl K5 mice until the mice reached 4.5-months of age 17. This difference may emerge because K14 expression starts earlier and occurs at higher levels than K5 expression 56. Moreover, a more severe phenotype has been observed for K14-cre conditional KO mice than for K5-cre conditional KO mice following deletion of Rac1 48, 57.

### Functional link between Cdc42 and the SPRR family in the regulation of epidermal barrier formation

In this study, we found that Cdc42 deficiency induced barrier defects and SPRR upregulation. We demonstrated that the expression of SPRRs during epidermal development is spatiotemporally dependent. However, the lack of Cdc42 in the basal layer not only led to increased expression of SPRR family members but also changed the location of their expression. The pattern of SPRR gene expression is closely associated with oral epithelial barrier formation 58. These observations raised two interesting questions: 1) What are the precise functions of SPRRs in epidermal development and epidermal barrier formation? 2) Is the overexpression of SPRR family members a molecular cause or effect of epidermal barrier dysfunction induced by Cdc42 deletion?

Two interesting findings of our study address these questions. First, the outside-in barrier, which was destroyed at E17.5, had recovered by P1. This phenomenon suggests that an increased abundance of SPRR proteins strengthens the barrier. In support of this possibility, Loricrin-deficient embryos exhibit a temporal delay in embryonic barrier competence until the upregulated expression of other components of the CE compensates 59. Furthermore, SPRR gene induction in cardiomyocytes responding to either biomechanical or ischemic stress renders a cardioprotective effect 42. Collectively, these data suggest that overexpression of SPRRs in response to stress occur as part of a protective repair response, as suggested by Segre 41. However, our results also showed that increased SPRRs led to increased proliferation that may result in transcription defects in tight junction proteins and barrier defects. Furthermore, reducing the abundance of SPRRs rescued the expression of junction protein expression *in vivo* and *in vitro*.

Considering that SPRR overexpression has multiple roles in the epidermal barrier, we propose that SPRR overexpression may have conflicting effects in epidermal barrier recovery: When the epidermal barrier is destroyed, SPRRs are overexpressed in the cornified envelope to repair the barrier structure. However, in other layers of the epidermis, the overexpression of these proteins can alter the junction proteins and destroy the epidermal barrier. To further investigate SPRR function *in vivo*, studies of conditional SPRR knockout mice or transgenic SPRR mice would be valuable. In addition, the mechanism of SPRR regulation of cell junctions remains to be addressed.

In this study, we found that the upregulation of SPRR genes induced by Cdc42 deletion was mediated by the p38-AP-1 signaling pathway. Promoter analysis of SPRR1A, 1B and 2A has shown that several transcriptional control elements are involved in differentiation-specific expression, including AP-1 47. In cardiomyocytes, MAPK triggers C/EBPbeta and AP-1 to regulate SPRR1A expression 42. In Clara-like H441 cells, Ras-MAPKKK1-JNK1 signaling regulates Jun and Fra1 to promote sprr1B expression 43. In S6 cells, the upregulation of SPRR1B expression stimulated by phorbol 12-myristate 13-acetate (PMA) is regulated by the MAPKK1- dependent AP-1 pathway 44. Our study reveals a new signaling mechanism for SPRR expression in keratinocytes.

### Implications of Cdc42 deficiency-induced epidermal barrier dysfunction in skin diseases

Many inflammatory skin diseases (e.g., psoriasis and atopic dermatitis) are accompanied by abnormal skin barrier function 60. Several SPRR family members are reportedly upregulated in skin disorders that feature skin barrier dysfunction 61-64. In this context, we propose that a keratinocyte-specific Cdc42-deficient mouse model may be relevant to inflammatory skin disease. In support of this hypothesis, the Cdc42-depleted epidermis contained an increased abundance of inflammatory cells (**Figure S8B**). Furthermore, localized barrier impairment could be considered a hallmark of incipient lesions in epidermal squamous cell carcinomas 65. Thus, our mouse model may also be relevant to skin cancers. Indeed, it has been reported that abnormal Cdc42 expression may contribute to the disruption of adhesion mechanisms in basal cell carcinoma 66.

## Conclusion

In this study, we used genetic techniques to investigate the role of Cdc42 in epidermal development. We found that Cdc42 deletion resulted in a profound skin barrier defect and early postnatal lethality. In addition, we demonstrated that Cdc42 plays crucial roles in regulating the homeostasis of epidermal stem cells, maintaining the balance between keratinocyte proliferation and differentiation, and preserving the integrity of the cell-cell junctions in epidermal development. The Cdc42-SPRR pathway may contain redundancy, which may correlate with epidermal barrier formation. This study significantly advances current understanding of the function of Cdc42 in epidermal development and epidermal barrier formation; this new knowledge will help maximize understanding of the molecular principles of skin physiology and disease development and support the search for new targets and theoretical options for the treatment of skin barrier-related disease.

## Supplementary Material

Supplementary methods, figures and table.

## Abbreviations

SPRRs: small proline-rich proteins
KO: knockout
WT: wild-type
K5: keratin 5
K14: keratin 14
TEWL: transepidermal water loss
K15: keratin 15
K19: keratin 19
CE: cornified envelope
TEM: transmission electron microscopy
SEM: scanning electron microscopy
Cdc42N17: Cdc42 dominant negative mutant
SH3BP: SH3 domain-containing protein
SC: stratum corneum
SS: stratum spinosum
SB: stratum basale
SG: stratum granulosum
