# Supplementary material for: Cdc42 Deficiency Leads To Epidermal Barrier Dysfunction by Regulating Intercellular Junctions and Keratinization of Epidermal Cells during Mouse Skin Development
Source: Theranostics. 2019 Jul 9;9(17):5065–84. doi: 10.7150/thno.34014 (PMC6691388; doi:10.7150/thno.34014)
Supplement: Supplementary file 1 — Supplementary methods, figures and table. [file thnov09p5065s1.pdf]

Supplementary material

Animals

The primer sequences for Cdc42$^{flox/flox}$ are as follows: P1, 5'-AGACAAAACAACAAGGTCCAGAAAC-3'; P2, 5'-CTG CCA ACC ATG ACA ACC TAA GTTC-3'. The primer sequences for K14-Cre are as follows: P1, 5'-TTC CTC AGG AGT GTC TTC GC-3'; P2, 5'-GTC CAT GTC CTT CCT GAA GC-3'; P3, 5'-CAA ATG TTG CTT GTC TGG TG-3'; and P4: 5'-GTC AGT CGA GTG CAC AGT TT-3'.

RNA-seq

Because the epidermis cannot be separated from the dermis in KO mice at E17.5, we performed RNA-seq with full-thickness skin at E17.5 and P1. Total RNA was extracted from the skin of WT and Cdc42 KO mice at E17.5 and P1 (n = 3 per genotype for each time point) using TRIzol according to the manufacturer's instructions (Invitrogen, USA). An additional DNase I digestion was performed to remove genomic DNA contamination. RNA quality and purity were checked using a NanoDrop (Thermo Fisher Scientific, Inc., USA). Then, total RNA samples were used to remove highly abundant ribosomal RNAs before sequencing.

The RNA was sequenced on an Illumina Genome Analyzer (HiSeq 2000) by Beijing Genomics Institute (Shenzhen, China). Library building and biological information analysis were performed by Total Genomics Solution Company (Shenzhen, China). Low-quality reads were discarded. The low-quality reads included over 50% of the sequence reads (including adapters) and were defined by quality

scores ≤ 10 and n > 5%. Ribosomal RNA sequences were filtered from the raw fragments. The remaining clean reads were aligned to the reference genome using hierarchical indexing for spliced alignment of transcripts (HISAT, http://www.nature.com/nmeth/journal/v12/n4/full/nmeth.3317.html).

The sequencing data obtained are available at the Gene Expression Omnibus website (http://www.ncbi.nlm.nih.gov/geo/) under accession number GSE55808. The data are also available at the Sequence Read Archive (SRA) website (https://submit.ncbi.nlm.nih.gov/subs/sra/) under accession numbers SRR6367468, SRR6367480, SRR6367477, SRR6367472, SRR6339686, SRR6339684, SRR6339685 and SRR6364983.

Analysis of differentially expressed genes

RNA-seq was performed, after which HTSeq v0.6.1 was used to count the number of reads mapped to each gene. Gene expression was calculated using the reads per kilobase per million mapped reads (RPKM) method. Genes with a false discovery rate (FDR)-adjusted p-value < 0.05 and fold change (FC) > 2 were considered to be significantly upregulated. Genes with an FDR-adjusted $p < 0.05$ and FC < 0.5 were considered to be significantly downregulated. Genes with FCs between 0.5 and 2 were considered to show no significant change in expression. Differentially expressed genes (DEGs) were analyzed by edgeR software (Release 3.1). All statistical analyses were performed with Pearson’s correlation coefficient based on transcript abundance (FPKM), which was used to measure the similarity of gene expression between samples.

Gene Ontology and pathway analysis

Significantly upregulated and downregulated genes were analyzed for enrichment of Gene Ontology (GO) terms (biological processes, molecular functions, and cellular components) using the Database for Annotation, Visualization and Integrated Discovery (DAVID). For GO term analysis, probabilities were evaluated with the Bonferroni correction, and FDR-adjusted *p*-values less than 0.05 were considered significant.

Supplementary figure legends

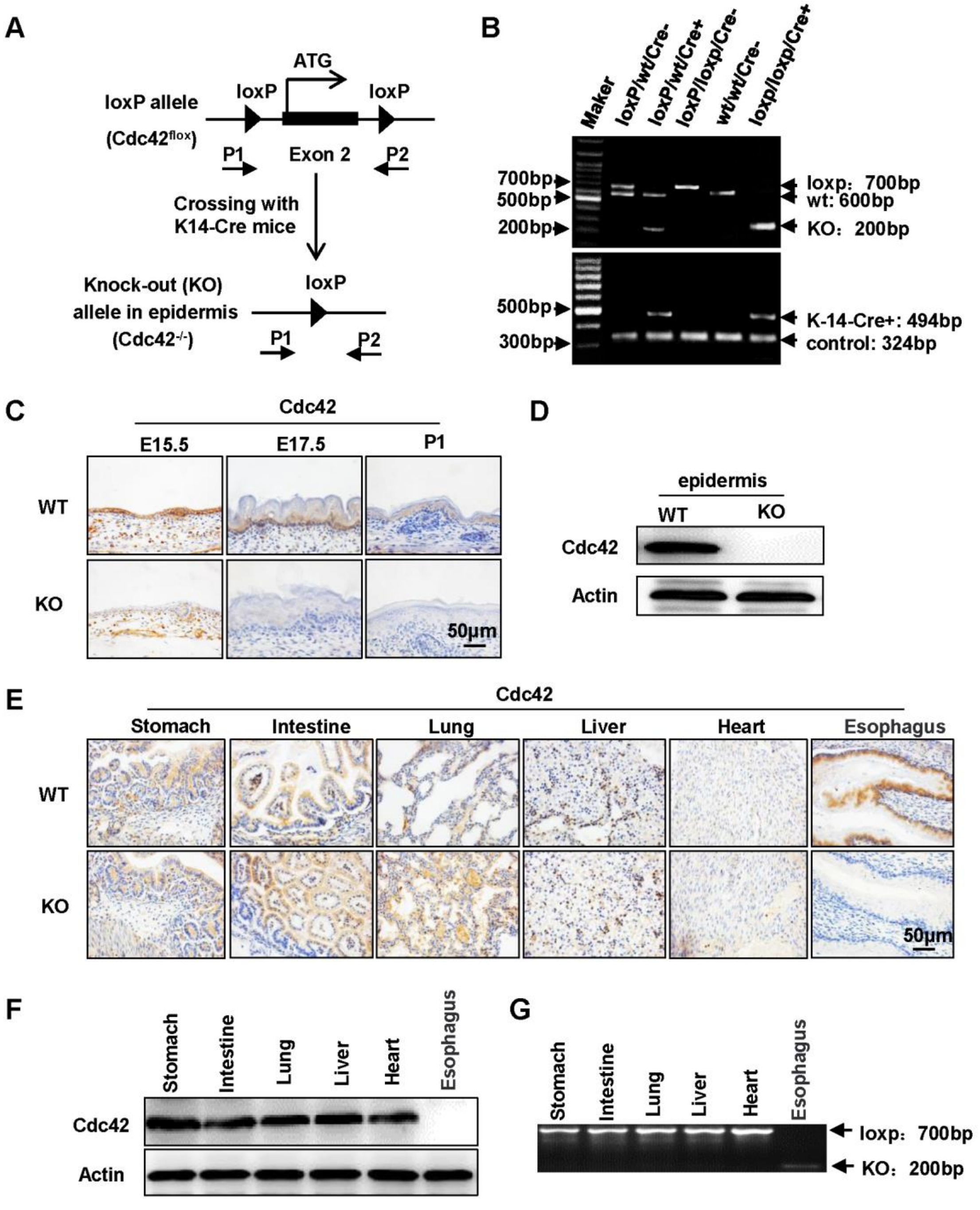

Figure S1. Generation of Cdc42-/- epidermis by Cre-LoxP recombination.

(**A**) The Cre-LoxP-mediated gene-targeting strategy to delete the Cdc42 allele in epidermis. Arrows indicate the PCR primers P1 and P2. (**B**) PCR verification of Cdc42 deletion in the epidermis. The 700-base-pair (bp) band represents the flox allele, the 600 bp band represents the WT (+) allele, the 200-bp band represents Cdc42 KO, the

494 bp band represents the Cre (+) allele, and the 324 bp band represents the internal positive control. The $Cdc42^{loxp/loxp/Cre+}$ mice were Cdc42 KO, and the $Cdc42^{wt/wt/Cre-}$/$Cdc42^{loxp/wt/Cre-}$/$Cdc42^{loxp/loxp/Cre-}$ were Cdc42 WT. (**C**) In immunohistochemistry experiments, Cdc42 expression gradually weakened from E15.5-E17.5 in KO mice compared to WT mice. After birth (P1), there was no Cdc42 expression in the epidermal basal cells of the KO mice. (**D**) The mouse epidermis and dermis were separated by neutral protease, after which the expression of Cdc42 in the epidermis was measured by WB in P1 WT and KO mice. (**E**) Immunohistochemistry detected the expression of Cdc42 in other important organ and tissues. (**F**) The expression of Cdc42 in the stomach, intestine, lung, live, heart and esophagus (epithelium) were detected by WB in P1 WT and KO mice. (**G**) PCR verification of Cdc42 deletion in the stomach, intestine, lung, live, heart and esophagus. n = 8. Scale bars: 50 μm.

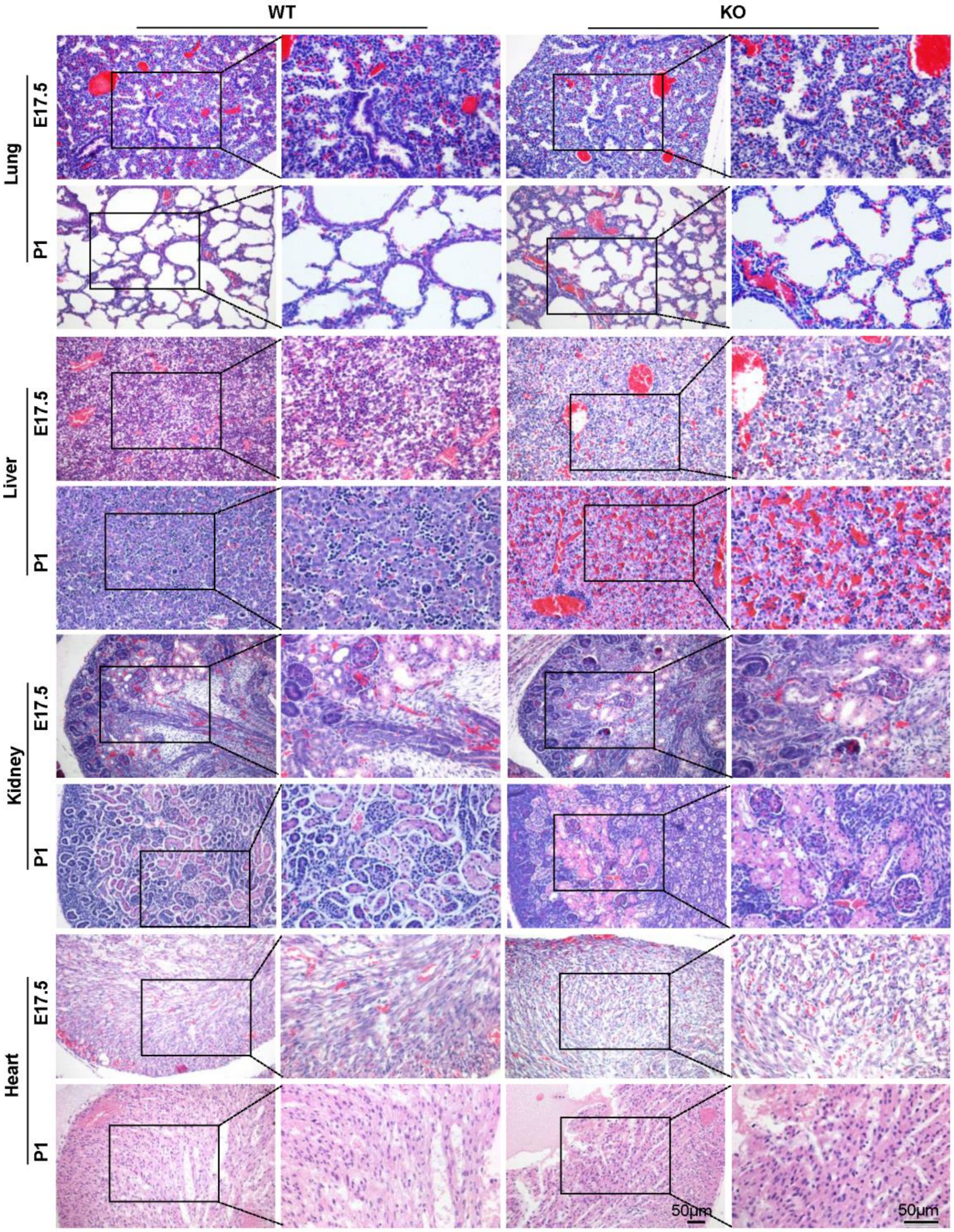

Figure S2. Tissue morphology of important organs in WT and KO mice.

Morphological observations of the liver, lungs, kidneys and heart of E17.5-P1 WT and KO mice (H&E staining). Scale bars: 50 μm.

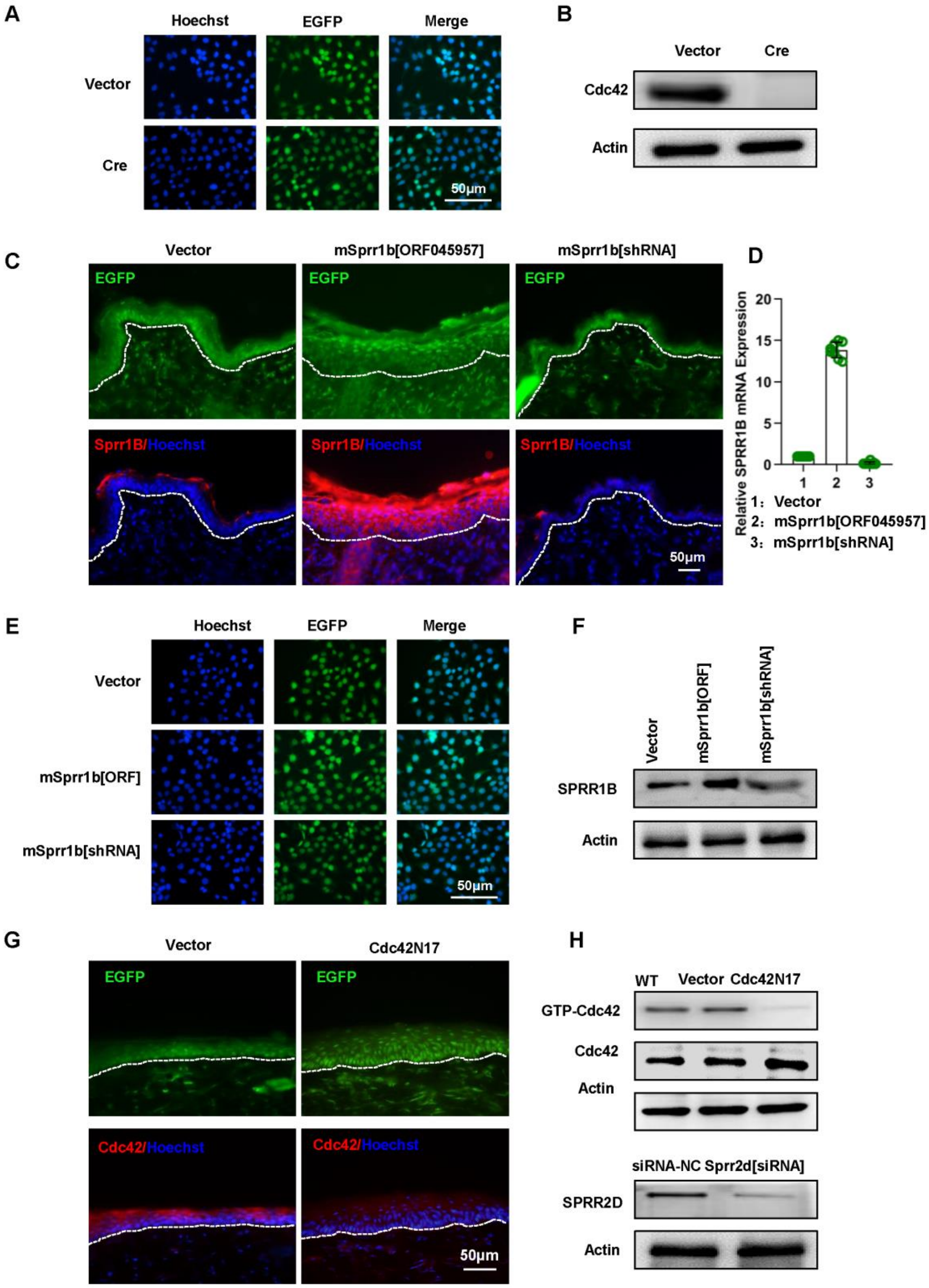

Figure S3. Detection of lentiviral infection efficiency in adult mouse skin and keratinocytes.

The infection efficiency (vector, plenti-Cre) was determined through green

fluorescence (**A**) and WB (**B**) in keratinocytes. The infection efficiency of lentiviral vector, mSprr1b[ORF045957], mSprr1b[shRNA] were determined by detecting green fluorescence in tissue sections with fluorescent microscopy (**C**) and qPCR (**D**) three days after lentiviral infection of WT mice. Scale bars: 50 μm. The infection efficiency (vector, Sprr1b[ORF] and Sprr1b[shRNA]) was also determined by examining green fluorescence (**E**) and WB (**F**) in keratinocytes. The infection efficiencies of the empty vector and Cdc42N17 vector lentiviruses were determined by detecting green fluorescence and the expression of Cdc42 in tissue sections with a fluorescence microscope after lentiviral infection of WT mice (**G**). Cdc42 activity (Cdc42-GTP) was assessed with a GTPase pull-down assay (**H**) three days after lentiviral infection. The interference efficiency of Sprr2d[siRNA]) was determined by WB (**I**) in keratinocytes.

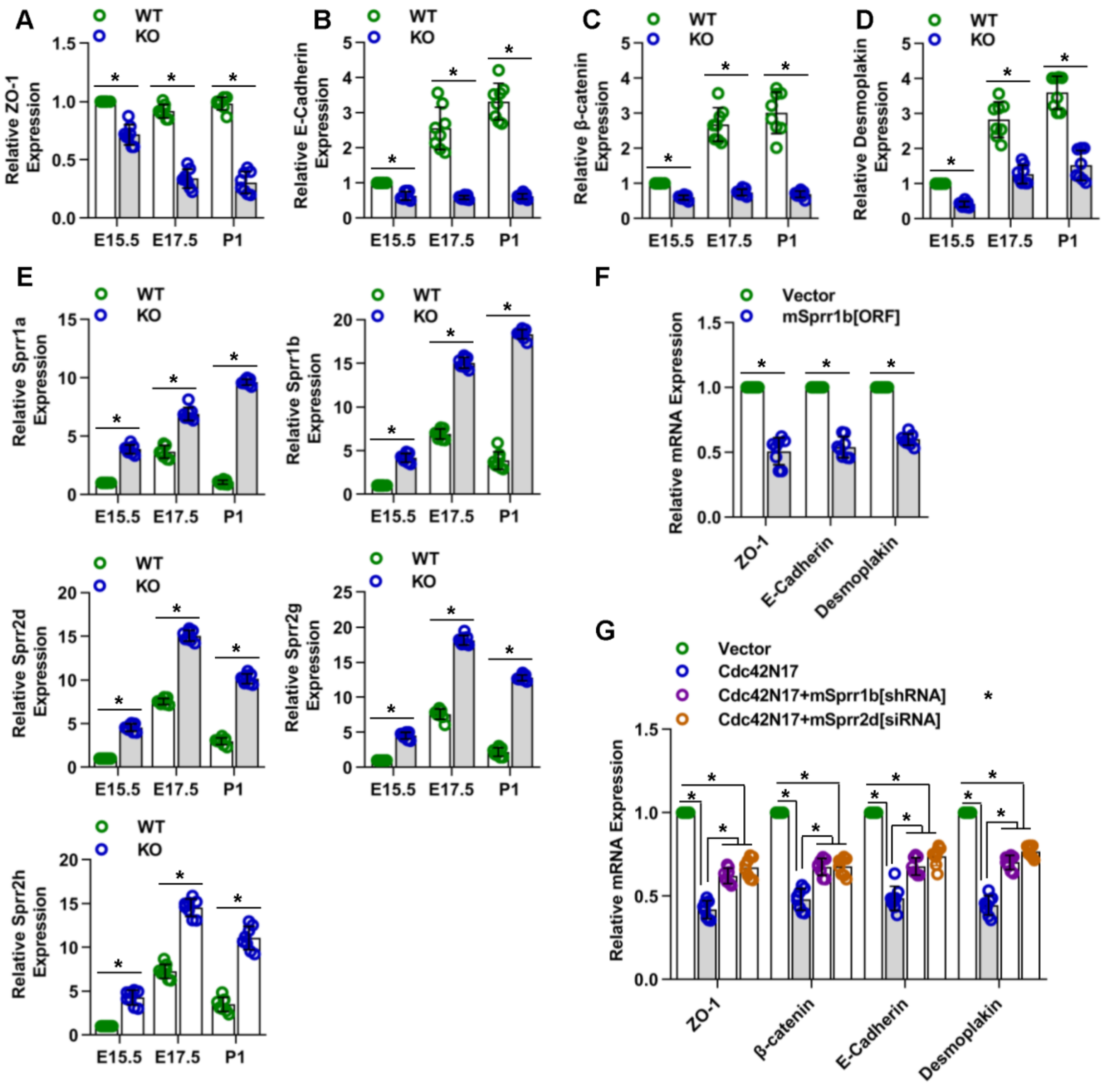

Figure S4. Protein expression was detected by RT-PCR.

The relative gene expression (normalized to GAPDH expression) of ZO-1 (**A**), β-catenin (**B**), E-cadherin (**C**) and desmoplakin (**D**) in Cdc42 KO and control mice. (**E**) Relative gene expression (normalized to GAPDH expression) of Sprr1a, Sprr1b, Sprr2g and Sprr2h in the Cdc42 KO epidermis. (**F**) Relative gene expression (normalized to GAPDH expression) of ZO-1, E-cadherin and desmoplakin in the epidermis of mSprr1b[ORF]-infected mice and vector-infected mice. (**G**) Relative gene expression (normalized to GAPDH expression) of S ZO-1, β-catenin, E-cadherin and desmoplakin in the epidermis of vector-infected mice, Cdc42N17-infected mice,

Cdc42N17+mSprr1b[shRNA] -infected mice and Cdc42N17+mSprr2d[siRNA] -infected mice. n=8. *p $<$ 0.05.

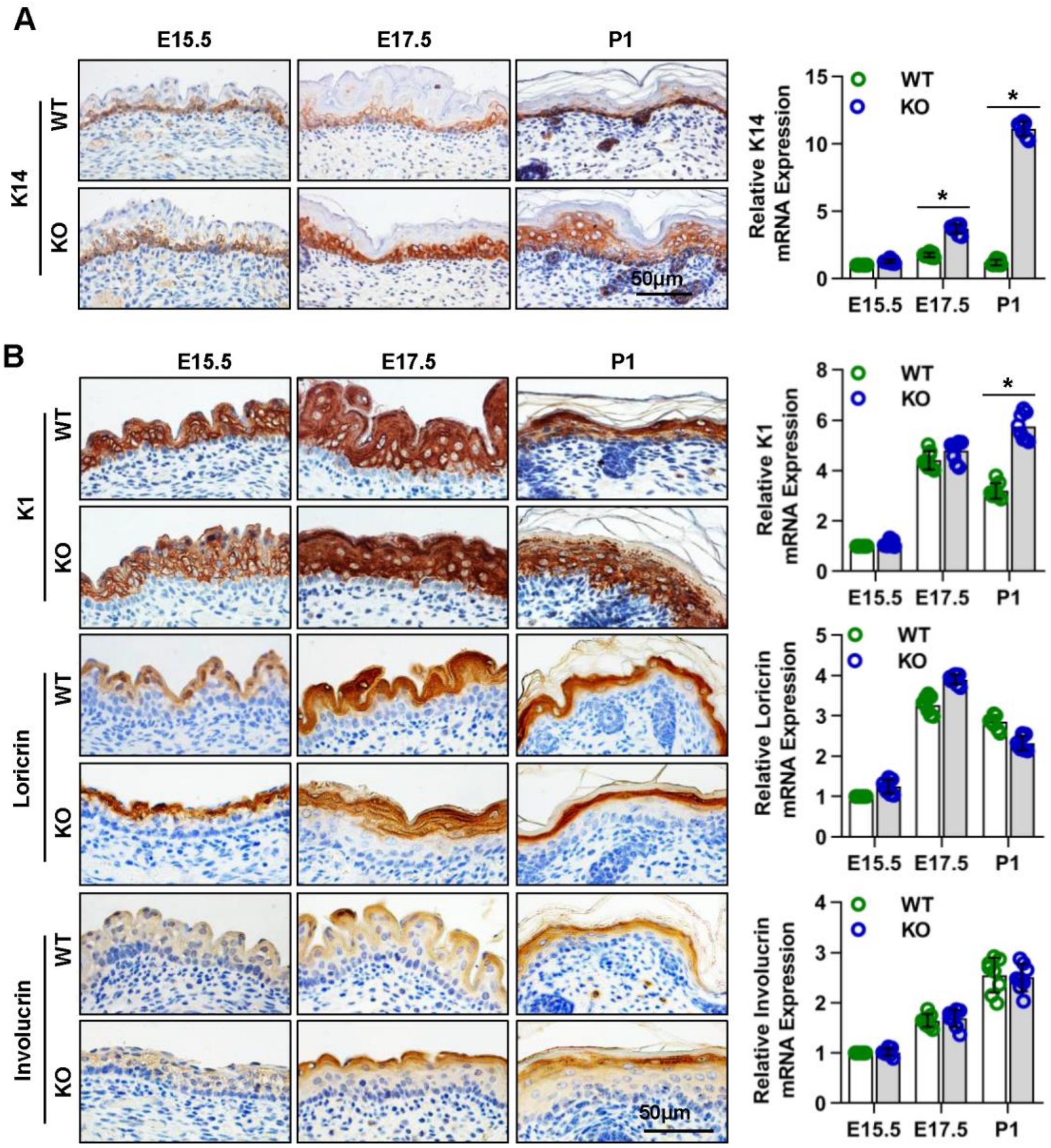

Figure S5. Severe alterations of the epidermis in the absence of Cdc42.

(**A**) Expression and distribution of K14 in Cdc42 KO and control mice by immunohistochemistry, and Relative gene expression (normalized to GAPDH expression) of K14 at E15.5, E17.5 and P1, n = 8. Scale bars: 50 μm. (**B**) Immunohistochemical detection of loricrin and involucrin at E15.5, E17.5 and P1 in the epidermis of Cdc42 KO and WT mice. Scale bars: 50 μm. Relative gene expression (normalized to GAPDH expression) of loricrin and involucrin. n = 8. *$p < 0.05$.

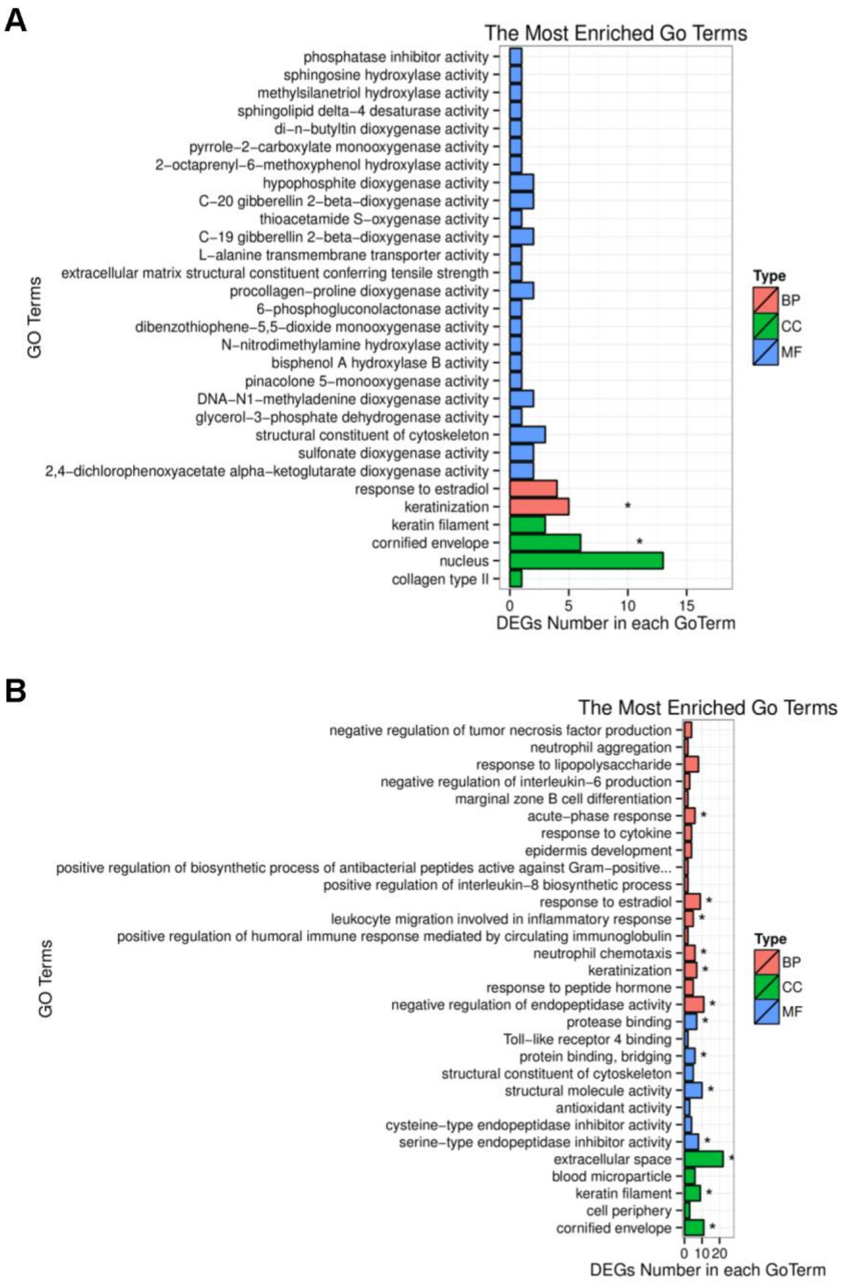

Figure S6. Cdc42 deficiency alters the expression of genes regulating keratinization and the CE.

(**A**) At E17.5, the most altered genes were involved with keratinization and the CE (n = 3). (**B**) At E19.5 (P1), once again, the most altered genes were involved with keratinization and CE. In addition, the GO terms “inflammatory response”, “defense response”, and “response to external stimulus” were enriched (n = 3).

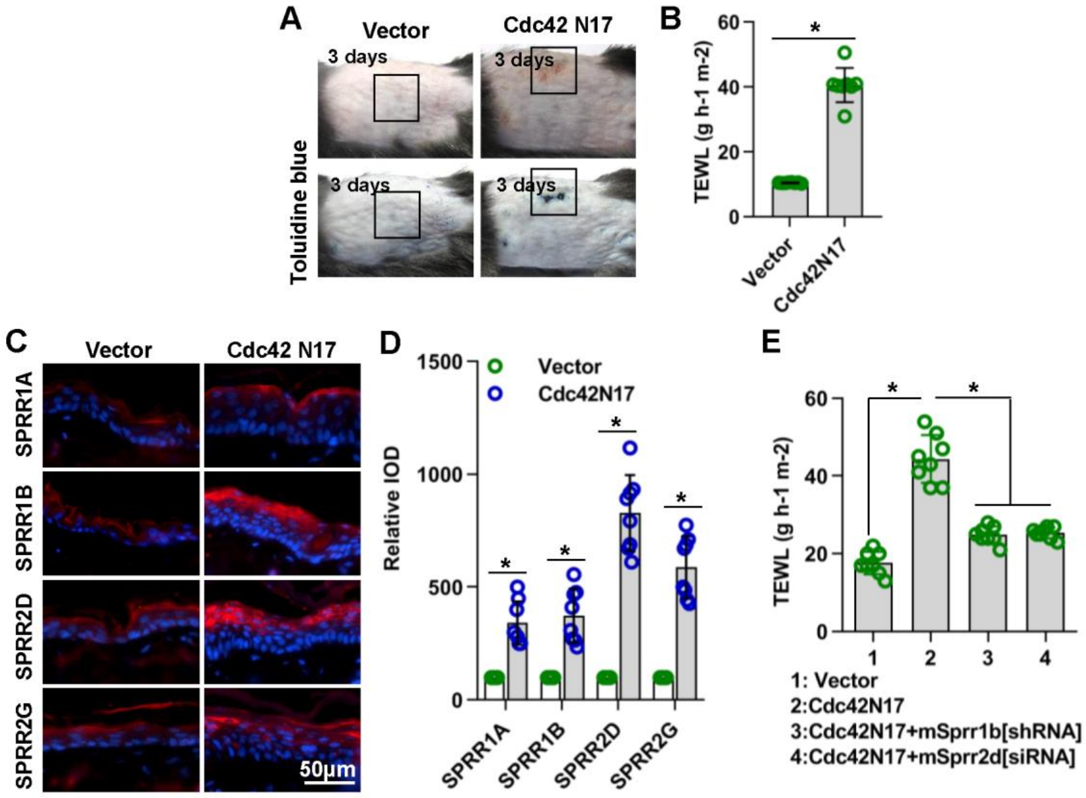

Figure S7. SPRR1B or SPRR2D is partially responsible for inside-out skin barrier dysfunction resulting from Cdc42 suppression in adult mice skin.

(A) The lentiviral vectors and Cdc42 N17 were applied to mouse skin. After 3 days, toluidine blue was used to assess the outside-in barrier function of the skin (n = 8). **(B)** TEWL was detected in the different groups (n = 8), *$p < 0.05$. The expression and distribution of SPRR1A, SPRR1B, SPRR2D and SPRR2G were determined by immunohistochemistry **(C)** and qPCR **(D)**, n = 8; *$p < 0.05$. Scale bars: 50 μm. **(E)** TEWL was detected in the different groups (n = 8), *$p < 0.05$.

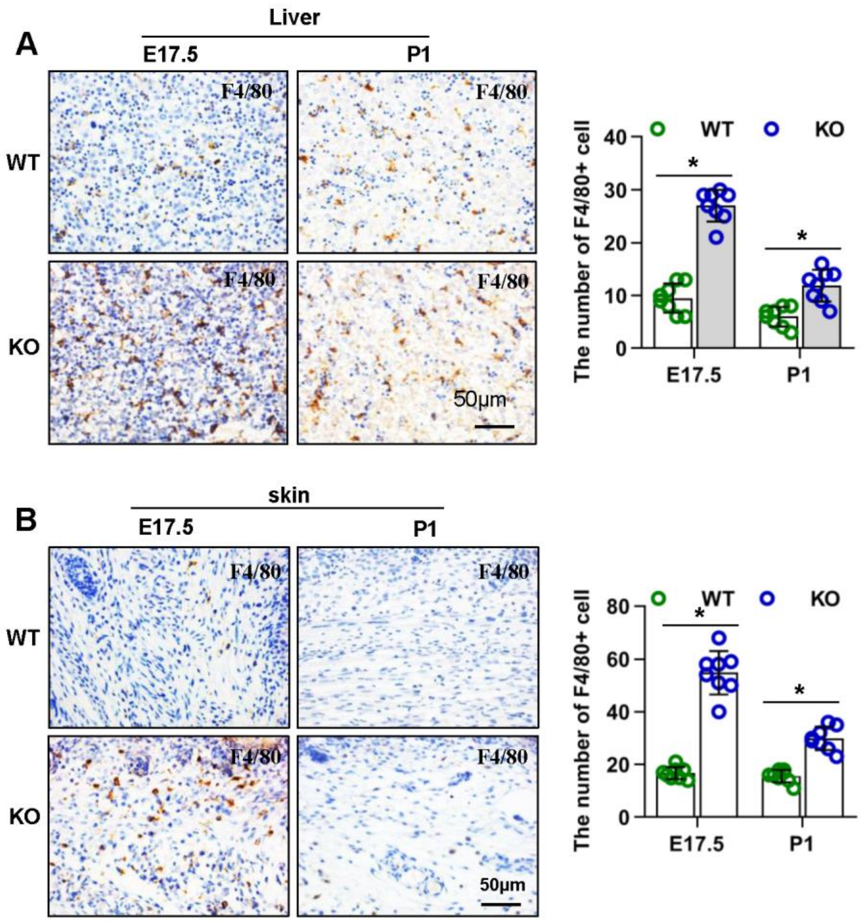

Figure S8. The abundance of inflammatory cells is increased in Cdc42-depleted epidermis.

F4/80^{+} cells were detected by immunohistochemistry and counted in the liver (**A**) and skin **(B)** of Cdc42 KO and WT E17.5 and P1 mice (n = 8 mice per genotype). *p **<** 0.05. Scale bars: 50 μm.

Table S1.

Primer combinations used for qPCR

	**Forward primer**	**Reverse primer**		
**Accession (gene)**	**Nucleotide sequence (5'-3')**	**Nucleotide sequence (5'-3')**	**Product size (bp)**	**Description**
NM_008084	AGGTCGGTGTGAACGGATTTG	TGTAGACCATGTAGTTGAGGTCA	123	*Mus musculus* glyceraldehyde-3-phosphate dehydrogenase (Gapdh)
NM_009265	TGAGCCATGTGTCTCACAAGT	GGGCTAGTATTGGAGTGACAGTT	350	*Mus musculus* small proline-rich protein 1B (Sprr1b)
NM_001165902	ATGGAGCCGGACAGAAAAGC	CTTGCCACTCAGGGAAGGA	108	*Mus musculus* catenin (cadherin associated protein), beta 1 (Ctnnb1)
NM_009864	CAGGTCTCCTCATGGCTTTGC	CTTCCGAAAAGAAGGCTGTCC	175	*Mus musculus* cadherin 1 (Cdh1)
NM_001163574	ACCACCAACCCGAGAAGAC	CAGGAGTCATGGACGCACA	710	*Mus musculus* tight junction protein 1 (Tjp1)
NM_023842	GCTGAAGAACACTCTAGCCCA	ACTGCTGTTTCCTCTGAGACA	107	*Mus musculus* desmoplakin (Dsp)
NM_008473	CTGTCTGTTCCCCTAGTGGC	GTCCGGGTTGTGGTGTCTAC	238	*Mus musculus* keratin 1 (Krt1)
NM_013707	CAGTATCCGATCTCTTCATGCG	GGGCTCACAGAAGGTTTCCTG	132	*Mus musculus keratin 14 (Krt14)*
NM_008508	GCGGATCGTCCCAACAGTATC	TGAGAGGAGTAATAGCCCCCT	262	*Mus musculus* loricrin (Lor)
NM_008412	ATGTCCCATCAACACACACTG	TGGAGTTGGTTGCTTTGCTTG	114	*Mus musculus* involucrin (Ivl)
NM_009264.2	TTGTGCCCCCAAAACCAAG	GGCTCTGGTGCCTTAGGTTG	237	*Mus musculus* small proline-rich protein 1A (Sprr1a)
NM_011470.2	AAGTGCCCACCTAAGAGCAA	AACATGGAGGGTGAAAGGTG	157	*Mus musculus* small proline-rich protein 2D (Sprr2d)
NR_003548.1	TCATTCCAAGAGCAGCAGTG	CTGGCATGGAGAAGGAAGAC	163	*Mus musculus* small proline-rich protein 2G (Sprr2g)
NM_011474.3	ATCTTCCCTCCAAAGCCATT	CTGAGCATCTGGGACAGTGA	168	*Mus musculus* small proline-rich protein 2H (Sprr2h)
